# PI3K/AKT Signaling Pathway Is Essential for Survival of Induced Pluripotent Stem Cells

**DOI:** 10.1371/journal.pone.0154770

**Published:** 2016-05-03

**Authors:** Amir M. Hossini, Annika S. Quast, Michael Plötz, Katharina Grauel, Tarik Exner, Judit Küchler, Harald Stachelscheid, Jürgen Eberle, Anja Rabien, Evgenia Makrantonaki, Christos C. Zouboulis

**Affiliations:** 1 Departments of Dermatology, Venereology, Allergology and Immunology, Dessau Medical Center, Dessau, Germany; 2 Department of Dermatology and Allergy, Skin Cancer Center Charité, Charité - Universitätsmedizin Berlin, Berlin, Germany; 3 NeuroCure Cluster of Excellence, Charité - Universitätsmedizin Berlin, Berlin, Germany; 4 Department of Biology, Chemistry, Pharmacy, Institute of Biology, Freie Universität Berlin, Berlin, Germany; 5 Berlin Institute of Health-Stem Cell Core Facility, Berlin, Germany; 6 Berlin-Brandenburg Center for Regenerative Therapies, Charité - Universitätsmedizin Berlin, Berlin, Germany; 7 Department of Urology and Berlin Institute of Urologic Research, Charité- Universitätsmedizin Berlin, Berlin, Germany; 8 Research Geriatrics Group, Charité - Universitätsmedizin Berlin, Berlin, Germany; 9 Department of Dermatology and Allergology, Universitätsklinikum Ulm, Ulm, Germany; University of Quebec at Trois-Rivieres, CANADA

## Abstract

Apoptosis is a highly conserved biochemical mechanism which is tightly controlled in cells. It contributes to maintenance of tissue homeostasis and normally eliminates highly proliferative cells with malignant properties. Induced pluripotent stem cells (iPSCs) have recently been described with significant functional and morphological similarities to embryonic stem cells. Human iPSCs are of great hope for regenerative medicine due to their broad potential to differentiate into specialized cell types in culture. They may be useful for exploring disease mechanisms and may provide the basis for future cell-based replacement therapies. However, there is only poor insight into iPSCs cell signaling as the regulation of apoptosis. In this study, we focused our attention on the apoptotic response of Alzheimer fibroblast-derived iPSCs and two other Alzheimer free iPSCs to five biologically relevant kinase inhibitors as well as to the death ligand TRAIL. To our knowledge, we are the first to report that the relatively high basal apoptotic rate of iPSCs is strongly suppressed by the pancaspase inhibitor QVD-Oph, thus underlining the dependency on proapoptotic caspase cascades. Furthermore, wortmannin, an inhibitor of phosphoinositid-3 kinase / Akt signaling (PI3K-AKT), dramatically and rapidly induced apoptosis in iPSCs. In contrast, parental fibroblasts as well as iPSC-derived neuronal cells were not responsive. The resulting condensation and fragmentation of DNA and decrease of the membrane potential are typical features of apoptosis. Comparable effects were observed with an AKT inhibitor (MK-2206). Wortmannin resulted in disappearance of phosphorylated AKT and activation of the main effector caspase-3 in iPSCs. These results clearly demonstrate for the first time that PI3K-AKT represents a highly essential survival signaling pathway in iPSCs. The findings provide improved understanding on the underlying mechanisms of apoptosis regulation in iPSCs.

## Introduction

Since the discovery by Yamanaka and co-workers [1] in the year 2006, induced pluripotent stem cells (iPSCs) are considered similar to embryonic stem cells (ESCs) with respect to pluripotency and quality. iPSCs are a promising ESCs alternative with similar differentiation potential, especially as a model to elucidate intractable disease mechanisms using original human cells of a patient [2–5]. These cells can differentiate into the three fundamental germ layers and thus give the researcher a great opportunity to develop over 200 human cell types in a dish for studying cellular processes, development of a tissue as well as underlying disease mechanisms, especially e.g. neurodegenerative or monogenic disorders [2,4,6,7]. Moreover, they could be used to replace disease-damaged cells and to investigate toxicological effects of drugs [8]. Unlike ESCs, development of iPSCs does not require any damage of embryos, but uses e.g. easy accessible skin cells like fibroblast cells. Another important advantage is that they can be generated directly from the patient's own cells, thereby circumventing the risk of rejection in case of cell replacement in patients and creating an effective treatment of each individual [9]. However, it is noteworthy that the undifferentiated iPSCs like ESCs have the propensity to develop into non-invasive tumours after transplantation in mice due to their unlimited proliferative potential [10]. These tumours exhibit histologically disordered structures of all three germ layers. Recently it has been reported that ESCs can be transformed after a long period in culture and are able to form aggressive tumors, so called teratocarcinomas [11,12]. As iPSCs behave similar to ESCs, it should be assumed that they possess the same propensity. Therefore, elucidating the poorly studied cell survival signalling pathways and death signalling pathways (e.g. apoptosis) in iPSCs is an urgent need which will provide new insight in their biological behaviour and allows us to generate an optimal autologous stem cell-replacement therapy without tumour formation.

Apoptosis failure due to multiple mutations promotes tumour development, whereas an uncontrolled excess of apoptosis is responsible for the development of neurodegenerative diseases such as Alzheimer's [13–15]. Proapoptotic signalling pathways possess far-reaching consequences for the fate of individual cells and are counter-regulated at multiple levels. The affected tissue is therefore well protected. Apoptosis can be triggered by the occurrence of proapoptotic and loss of antiapoptotic signals. Biological homeostasis and physiological functions in the tissue are based on the balance of these two regulatory limbs. The proteins of the Bcl-2 (B cell lymphoma oncogene 2) family are important decisive regulators of apoptosis [15,16]. For many cellular systems, it is assumed that the molar ratio between proapoptotic (BAX, BAK, BCL-X_S_, BAD, PUMA, and others) and anti apoptotic (BCL-2, Bcl-X_L_, MCL-1, A1, etc.) members of the Bcl-2 family can decide on the susceptibility to apoptotic signals [17]. In addition to these, caspases, a family of cysteinyl aspartate-specific proteases, are central mediators of apoptotic and inflammatory processes. They conduct death signals and are capable to cleave a number of proteins known as death substrates to enhance the apoptotic signals and to complete the apoptotic process [18]. Surprisingly both, the apoptotic initiator caspase-8 and the effector caspase-3 are essential for iPSC reprogramming [19].

The cell survival signaling PI3K (phosphoinositide 3-kinase) pathway plays a crucial role for mitogenesis, proliferation, prevention of apoptosis, maintenance of multipotency in mesenchymal stem cells (MSCs) and pluripotency in ESCs [20,21]. It can be activated exogenously by growth factors and cytokines and endogenously by the constitutively active Ras protein. Both apoptosis and survival of cells can be controlled by proteins such as AKT/PKB (protein kinase B) involved in the PI3K pathway [21]. Phosphorylated AKT enhances survival and inhibits apoptosis by phosphorylation and inactivation of several target proteins including the proapoptotic BAD and the tumour suppressor p53, a regulator of bcl-2 and bax genes [22,23]. Moreover, several studies have recently reported that the AKT/PKB signaling pathway is involved in cell survival in various cancers such as colon carcinoma, glioma, melanoma, breast and liver carcinoma due to the abnormal activation of the downstream gene AKT [24–26]. Several sudies demonstrated that the PI3K signalling pathway is essential and crucial for proliferation, survival and maintance of pluripotency in mouse embryonic stem cells [21].

To date the role of the PI3K-AKT signalling pathway and its cellular response in human iPSCs have been poorly defined. Known is, however, that PI3K-AKT signalling is involved in early steps of the reprogramming process in order to switch from oxidative phosphorylation to glycolysis [27,28]. PI3K signalling parallel to upregulation of glycolytic gene expression during reprogramming processes shows in particular that AKT activates two key glycolytic regulators, AS1060 and PFKB2 [29]. Another study reported that PS48, an activator of PI3K-AKT, is able to enhance reprogramming by upregulating glycolytic genes [30]. Also in this context, Chen et al., [31] demonstrated an enhancement of somatic reprogramming in mice by application of LY294002, another PI3K inhibitor. Therefore, understanding of PI3K-AKT signalling in iPS cells, especially regarding their similarity to cancer cells, is of great importance.

In this study we used the small molecule PI3K inhibitor wortmannin and the AKT inhibitor MK-2206 to investigate the molecular consequences of this prominent target signalling pathway in three different iPSC lines which were derived from skin fibroblasts of an Alzheimer patient, from normal human fibroblasts and from human foreskin fibroblasts, respectively. The three iPS cell lines used in this study were generated by three different reprogramming approaches. Additionally, we controlled four other relevant pathways using three small molecule inhibitors and one apoptosis inducer. All of these pathways are dysregulated in cancer due to mutations of the genes involved. The Pan-RAF inhibitor L-779,450 which suppresses cell proliferation in melanoma cell lines both with mutated B-Raf and with wild-type B-Raf [32], BMS-345541, an IKK-2 inhibitor that addresses the NF-kappaB pathway and has antiinflammatory properties and Aurora kinase an inhibitor MLN-8237 (AKA-I), which inhibits a member of the novel family of Aurora serine/threonine kinases crucial for cell cycle control [33]. As apoptosis inducer we used Tumor Necrosis Factor Related Apoptosis Inducing Ligand (TRAIL) which is important for the immune system. TRAIL belongs to the TNF / TNFR (Tumor Necrosis Factor Receptor) superfamily. Its mission is to inactivate target cells by the extrinsic pathway of apoptosis, but most cancer cells are resistant to this agent [34–36]. Furthermore we tested the p53 pathway using pifithrin-alpha (PFT-alpha), a pharmacological inhibitor of p53 [37]. The aim of this study was to determine whether there are differences regarding the apoptotic behaviour on the cellular and molecular level between generated iPSCs, parental fibroblast cells and iPS derived neurons after treatment with different inhibitors of crucial signalling pathways involved in cancer. Overall, striking differences could be shown in the reaction of the iPS cell lines to small molecules versus the original skin fibroblasts they were derived from or versus terminally differentiated neurons derived form these iPSCs.

## Materials and Methods

### Ethics statement

Full-thickness skin biopsy was resected from the forearm of a patient undergoing surgery. A small skin biopsy (6 mm) of a not sun-exposed body region (inside upper arm) was removed. The procedure was performed under local anesthesia. The study protocol has been approved by the Charité Universitätsmedizin Berlin ethics committee. Ethical agreement was preliminarily obtained from the guardian of the participant including written informed consent.

### Cell culture

For this study we used frozen iPSCs generated from skin cells taken from an Alzheimer patient as described in a previous study [5]. Briefly, after two years of storage in liquid nitrogen the iPSCs were quickly thawed at 37°C and cultured in hESCs medium containing KO-DMEM supplemented with 20% knockout^TM^ serum replacement, 1% nonessential amino acids, 1% L-glutamine, penicillin/streptomycin, 1% sodium pyruvate, 0.1 mM beta-mercaptoethanol (all from Invitrogen, Karlsruhe, Germany) and 8 ng/ml basic fibroblast growth factor (bFGF) (Preprotech, Rocky Hill, NJ, USA) on dishes coated with matrigel (BD; San Diego, CA). Subsequently iPSCs were cocultured with mitomycin-c treated feeder cells (CF1 MEF, p3, Cell System, Troisdorf, Germany) for 5 weeks. When showing their typical morphology, the iPSCs were passaged manually and transferred into a new matrigel-coated feeder-free plate. The cells were grown in 5% CO_2_ with 95% humidity in mTeSR1 medium complete kit (STEMCELL Technologies SARL, Cologne, Germany). The medium was changed daily, iPSCs were passaged every 6 days with 5 U/mL dispase (STEMCELL Technologies SARL) at 37°C for 7–10 min, and spontaneously arising differentiated cells were removed manually under a microscope. In our study, we used the following 5 small molecules, namely wortmannin (0.5–4μM, PI3K inhibitor, Santa Cruz Biotechnologies, Santa Cruz, CA, USA), MK-2206 (2μM, AKT inhibitor, (8-,4-,1- aminocyclobutyl)phenyl)-9-phenyl-(1,2,4)trizolo(3,4-f)(1,6naphthyridin-3(2H)-one; Sellekchem, Munich, Germany), L-779,450 (4μM, pan-RAF inhibitor, Merck, Darmstadt, Germany;), BMS-345541 (4μM, IKK-2 inhibitor that addresses the NF-kappaB pathway; Sigma-Aldrich, Taufkirchen, Germany), MLN-8237 (50nM, Aurora A-kinase inhibitor, Seleckchem, Munich, Germany), and the death ligand TRAIL (10ng/ml, Alexis, Gruenberg, Germany). For caspase inhibition, iPSCs were pretreated for 1h with 10 μM of the pancaspase inhibitor QVD-oph (MP Biomedicals, Solon, OH, USA).

Furthermore we used two other iPSC lines. iPSC line BIHi001-A was derived from human foreskin fibroblasts (HFF; CCD-1112Sk, American Type Culture Collection (ATCC) were reprogrammed using Sendai virus vectors expressing Oct3/4, Sox2, Klf4, and c-Myc (CytoTune-iPSC reprogramming kit, Life Technologies)). The iPSC line BIHi004-A was derived from normal human fibroblasts (NHDF-Ad-Der Fibroblasts, Lonza, Basel, Switzerland) using episomal vectors expressing Oct3/4, Sox2, Klf4, L-Myc, Lin28 mp53DD, and EBNA1 (Epi5 Episomal iPSC Reprogramming Kit, Life Technologies). Both iPSC lines were cultivated in Essential 8 Medium (E8, Thermo Fisher Scientific). Cells were tested for absence of remaining reprogramming vectors, expression of the pluripotency markers OCT4, SSEA4, TRA-1-60, TRA-1-81, SOX2 and differentiation capacity into cells of the three germ layers. Detailed information and characterization data of the lines is available in the hPSCreg database (http://hpscreg.eu/cell-line/ BIHi001-A, http://hpscreg.eu/cell-line/ BIHi004-A). For p53 inhibition, all three iPSCs were pretreated for 2h with 4μM, 10μM and 50μM PFT-alpha (Sellekchem).

### Alkaline phosphatase analysis and immunofluorescence staining

Alkaline phosphatase (AP) activity was visualized by using the commercially available AP staining kit (AP-red, Millipore, cat. no. SCR004, Schwalbach, Germany; AP-blue, Sigma Aldrich, cat. no. AB0300), according to the manufacturer's instructions. In order to characterize AD-iPSC colonies in terms of pluripotency properties, the cells were stained employing immunocytochemistry. Cells were first fixed in phosphate-buffered saline (PBS) containing 4% paraformaldehyde (Science Services, Munich, Germany) for 20 min at room temperature, subsequently washed twice with PBS without Ca^2+^ and Mg^2+^and finally blocked with 10% FBS serum (Vector Laboratories, Loerrach, Germany) and 0.1% Triton X-100 (Sigma-Aldrich) in PBS. A prominent stem cell marker was used as primary antibody, NANOG (1:100, Abcam, cat. no. ab62734, Cambridge, UK), followed by an Alexa-488-conjugated secondary antibody (1:300, Invitrogen, cat. no. A11001). Nuclei were counter-stained with 4′,6-Diamidin-2-phenylindol (200 ng/ml, DAPI, Invitrogen) and visualized using fluorescence microscopy.

### Quantitative real-time polymerase chain reaction and data analyses

Total RNA was isolated using the RNeasy Mini Kit (Qiagen, Germantown, MD, USA,) according to the manufacturer's instructions. In each case 1 μg RNA was used to generate cDNA using SuperScriptIII First Strand Synthesis System for RT-PCR (Thermo Fisher Scientific, Waltham, USA). Quantitative real-time PCR (RT-qPCR) was performed with SYBR®Green PCR Master Mix (Applied Biosystems, Foster City, CA, USA) on a Quant Studio 6 Real-Time PCR machine and analyzed using the QuantStudio™ Real-Time PCR Software (Applied Biosystems, Foster City, CA, USA) and Microsoft Excel. Samples were normalized to HPRT (Hypoxanthine Phosphoribosyltransferase) as housekeeping gene and analyzed using the deltadeltaCt method. All primer sequences are provided in S1 Table. For detection of pluripotency-associated genes in AD-iPS, BHIi001-A-iPS and BHIi004-A-iPS, the undifferentiated hESC line WAe001-A (H1) (positive control) and Human Foreskin Fibroblasts (HFF, negative control) were used. All qPCR data are presented as mean ± s.e.m. of at least three replicates.

### Neuronal differentiation

Neuronal cells were generated according to a recently published protocol, [38]. Briefly, AD-iPSCs were cultured in mTeSR1 medium (STEM CELL) on 6 well plates coated with Matrigel (BD Bioscience) for two weeks. Induction of neuronal cells was performed by adding 10 μM SB431542 (SB, TGF-beta receptor inhibitor) and 1 μM PD0325901 (PD, MEK1/2 inhibitor) (both from Sigma-Aldrich) in the absence of bFGF (Preprotech; Rocky Hill, NJ) [38]. The cells were grown for additional six weeks and the medium was changed daily. Furthermore, induced neurons from BIHi001-A and BIHi004-A were generated by forced expression of the transcription factor Ngn2 as previously described [39]. Briefly, iPSCs were transduced with lentiviral vectors expressing Ngn2 and rtTA as well as a puromycin resistance gene. Ngn2 expression was induced by the addition of doxycyclin (2 μg/μl) on day 0 and succesfully infected cells were selected by addition of puromycin (1μg/ml) for 2 days. To avoid contamination of the following assays with mouse astrocytes, induced neurons were cultured suspended above an astrocyte feeder layer [40] The obtained neuronal cells were treated with wortmannin after eight weeks (AD-iPSCs) or after 21 days (BIHi001-A and BIHi004-A) and used for Western blotting and measurement of apoptosis by FACS analysis.

### Apoptosis detection and cell cycle analysis

For quantification of apoptosis and cell cycle arrest, cell cycle analyses were performed [17]. In brief, treated cells (usually 1 h to 24 h) were harvested using accutase (Millipore, Schwalbach, Germany) and stained for 1 h with propidium iodide (200 mg/ml; Sigma-Aldrich). Sub-G1 fractions corresponding to cells with fragmented DNA were quantified by flow cytometry (FACS Calibur, BD Bioscience, Bedford, MA, USA; 10,000 cells gated, FL3H, and MACSQuant VYB, Miltenyi Biotec, Bergisch Gladbach, Germany; 10,000 cells counted). Apoptosis induction and cell cycle arrest of treated cells were compared to untreated controls. Apoptosis was subsequently quantified by using a cell death detection enzyme-linked immunosorbent assay (ELISA) (Roche Diagnostics, Mannheim, Germany), which detects mono- and oligonucleosomes formed in apoptotic cells according to a protocol described previously [41]. We diluted the samples 1: 500 so that a slow colour reaction could take place, since the induction of apoptosis with wortmannin is very strong. Relative apoptotic rates were calculated as the ratio of ELISA values of wortmannin treated cells to non-treated cells. Each assay consisted of triple values, and at least three independent experiments were performed.

For visualization of typical features of apoptosis, we treated iPSCs with wortmannin for 1h and fixed the cells in 4% paraformaldehyde (Science Services) for 20 min at 4°C in 24 well plates and washed them once with PBS. 1 μg/ml Hoechst-33258 dye (Sigma-Aldrich) was added for 20 min at room temperature after which cells were washed again with PBS. Cells were examined by fluorescence microscopy. Apoptotic cells were identified by condensed and fragmented nuclei. For further confirmation of apoptosis induction we used the cell death detection kit.

### Determination of mitochondrial membrane potential and reactive oxygen species formation

Mitochondrial membrane potential was determined with the fluorescent dye TMRM^+^ (1 mM; tetramethylrhodamine methyl ester perchlorate; Sigma-Aldrich). Cells were treated with wortmannin for 1–24 h, harvested by accutase and stained with TMRM^+^ for 15 min at 37°C. Then, iPSCs were measured in PBS by flow cytometry in a FACS Calibur (1x 10^4^ gated cells, FL2H). For determination of intracellular reactive oxygen species (ROS), cells were treated for 0.5–24h. After using accutase, cells were stained for 30min with the fluorescent dye 20,70-dichlorodihydrofluoresceindiacetate (15mM; H2DCFDA, Molecular Probes, Invitrogen, Eugene, OR, USA) and measured in PBS by flow cytometry in a FACS Calibur (10^4^ gated cells, FL1H). For ROS scavenging, cells were pretreated for 1h with 200–400μM alpha-tocopherol (vitamin E) (Fluka, Steinheim, Germany). As positive control, cells were treated with 200mM H_2_O_2_ for 1h.

### Western Blot

Human iPSCs and their corresponding fibroblasts as well as iPSC-derived neurons were detached from the surface of the cell culture dish by incubation with accutase. For protein extraction, cells were harvested within 1–24 h after wortmannin treatment and lysed in RIPA buffer supplemented with complete protease inhibitor and phosphatase inhibitor cocktail (Roche, Penzberg, Germany). Extracts were homogenized and centrifuged at 12000×g for 10 min. SDS–polyacrylamide gel electrophoresis (PAGE) and Western blot analysis of total proteins were performed as previously described [42]. The following antibodies were used: primary antibodies from Cell Signaling (Danvers, MA, USA), cleaved caspase-3 (3664, rabbit, 1: 1000), P-BAD (9292, rabbit, 1: 1000); primary antibodies from Santa Cruz Biotech, Bcl-2 (sc-7832, rabbit; 1: 200), BAX (sc-493, rabbit, 1: 200), PUMA (sc-374223, rabbit, 1: 1000), p53 (sc- 6243, rabbit, 1:200) and from Novus light chain 3A (LC3A) (NB100-2331, rabbit, 1:50). As loading control for the same amount of proteins primary antibodies for alpha-actin and beta-tubulin from the Loading Control Antibody Sampler Kit from Cell Signaling (4670, rabbit, 1:500) were used. After incubation with a peroxidase-labelled anti-rabbit and anti mouse secondary antibody (1:5000, Dako, Hamburg, Germany), antigen–antibody complexes were detected by ECL Western blotting detection reagents (Peqlab, Erlangen, Germany). One to 15 min exposures were analysed with the Fusion FX7 imaging system (Peqlab).

### Statistical analyses

Assays consisted of triplicates, and at least two independent experiments were performed. Mean values and standard deviations (SD) were calculated by enclosing all individual values of the independent experiments (at least 6 values). Statistical significance was proven by Student’s t-test (normal distribution) and p-values of < 0.05 were considered statistically significant.

## Results

### Cultivation of thawed iPSCs and their characterization in terms of pluripotent properties

**Fig 1:**
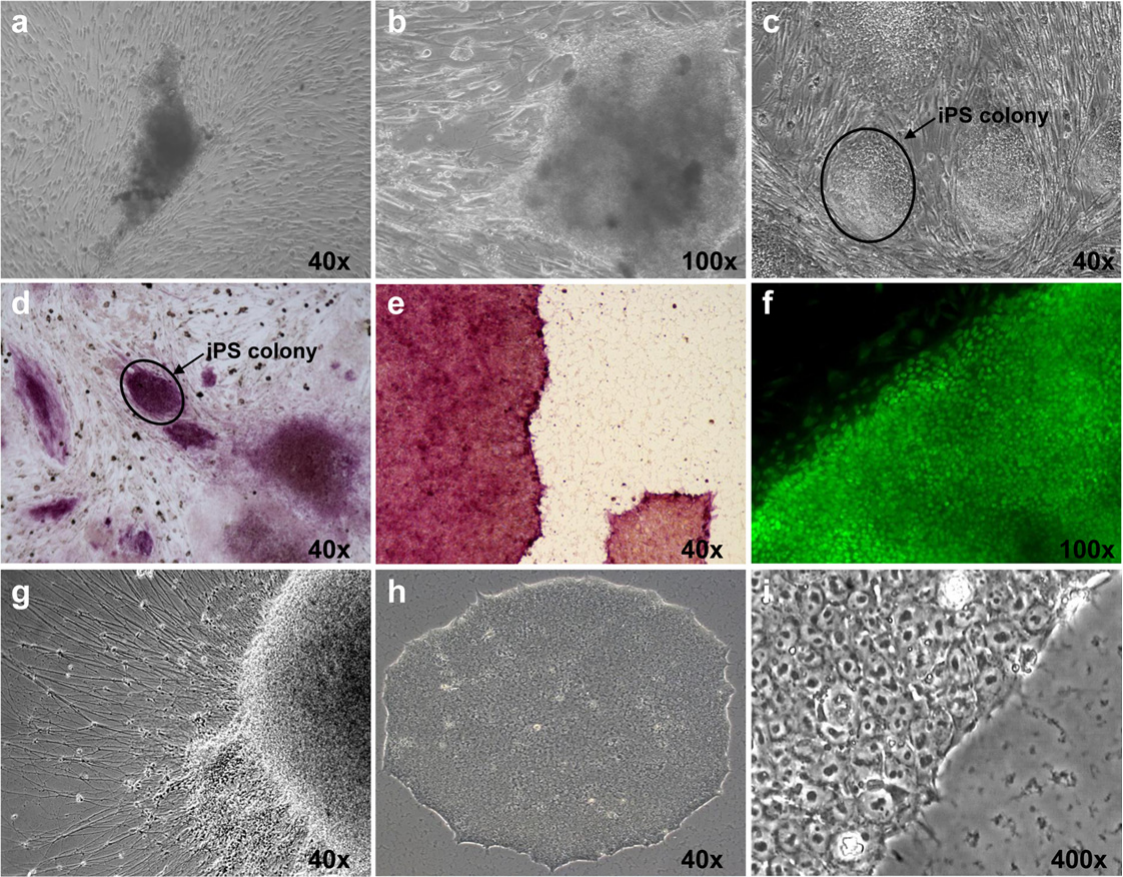
Cultivation and characterization of AD-iPSC colonies for pluripotency. (a) AD-iPSCs one week after thawing on feeder cells. (b) AD-iPSCs two weeks in culture in high magnification. (c) Typical AD-iPSC morphology appears 4 weeks after thawing. (d-e) After two years freezing in liquid nitrogen the AD-iPSCs were positive for pluripotency associated alkaline phosphatase (AP) staining both on feeder cells and in a feeder-free system. (f) The AD-iPSCs were positive for embryonic stem cell markers such as NANOG. (g) Spontaneous neuronal differentiation of AD-iPSCs in vitro. (h-i) Typical AD-iPSCs morphology in low and high magnification.

Human AD-iPSCs arised from skin fibroblasts through gene delivery using a retroviral vector containing the four transcription factors OCT4, SOX2, KLF4 and C-MYC as reported in a previous study [5]. One iPSC clone was thawed quickly after two years liquid nitrogen storage and was co-cultured with feeder cells. The iPSCs were tested positive for alkaline phosphatase activity and the stem cell specific protein Nanog, giving evidence for pluripotent stem cell properties. We developed neuronal cells from iPSCs as described in the Methods section to compare the effect of wortmannin on differentiated and undifferentiated cells. After five weeks co-culture with feeder cells, iPSC clones we manually picked and subcultured feeder-free in mTeSR1 medium. Spontaneously differentiated cells were removed manually under a microscope. The thawn iPSCs exhibited hESC-like morphology (Fig 1). Two additional Alzheimer-free iPSC lines used in this study were subcultivated in E8 medium which was replaced by mTeSR1 one day before treatment. Both BIHi004-A and BIHi001-A-iPSCs were positive for pluripotency gene expression of OCT3/4, Sox-2, Nanog detected by RT-qPCR analysis (S1A Fig) and showed a high alkaline phosphatase ezyme activity (S1B Fig), indicating their pluripotency. Furthermore these cells could differentiate into neurons as described [39,40].

### High basal cell death in iPSCs is caspase-dependent

**Fig 2:**
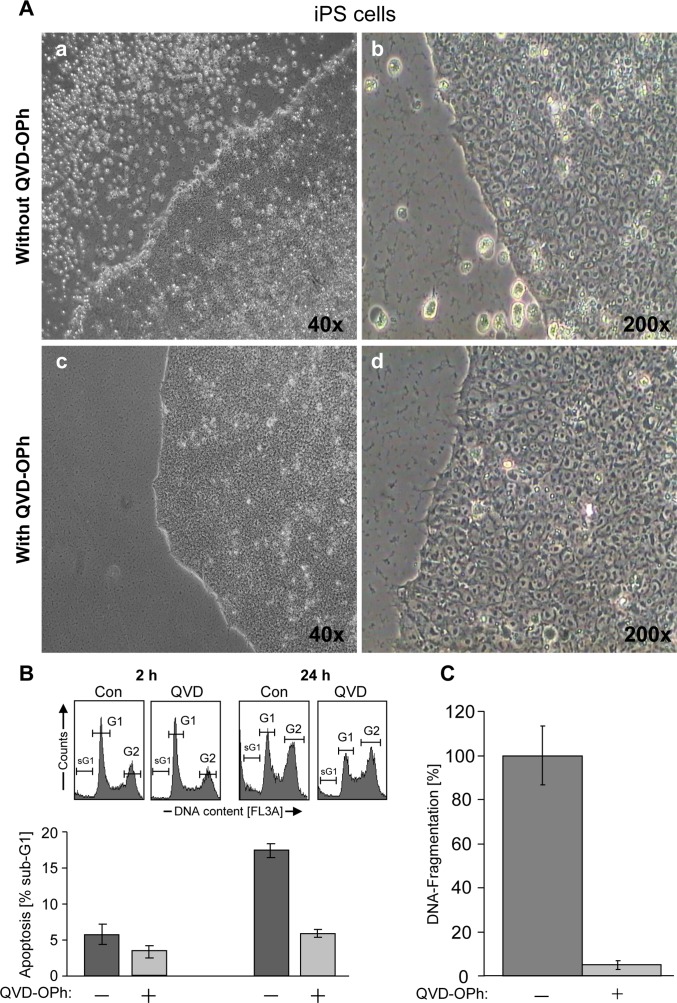
High basal spontaneous apoptosis in AD-iPSCs is caspase dependent. (A) Pictures were taken 24 h after daily changing medium. (a-b) AD-iPSCs as a control with a lot of dead cells in the supernatant after 24 h in two different magnifications in contrast to iPSCs pretreated with 10 μm caspase inhibitor (c-d). (B) Employing FACS analysis the apoptosis (percentage of sub-G1 cells) was determined by cell cycle analysis in AD-iPSCs pretreated with 10 μm caspase inhibitor (QVD-oph) for 2 h and 24 h. After pretreatment of AD-iPSCs with QVD-oph basal apoptotic cells decreased significantly compared to iPSCs without QVD-oph. (C) In support to our FACS analysis, apoptosis was quantified by determination of DNA fragmentation using ELISA cell death detection kit.

**Fig 3:**
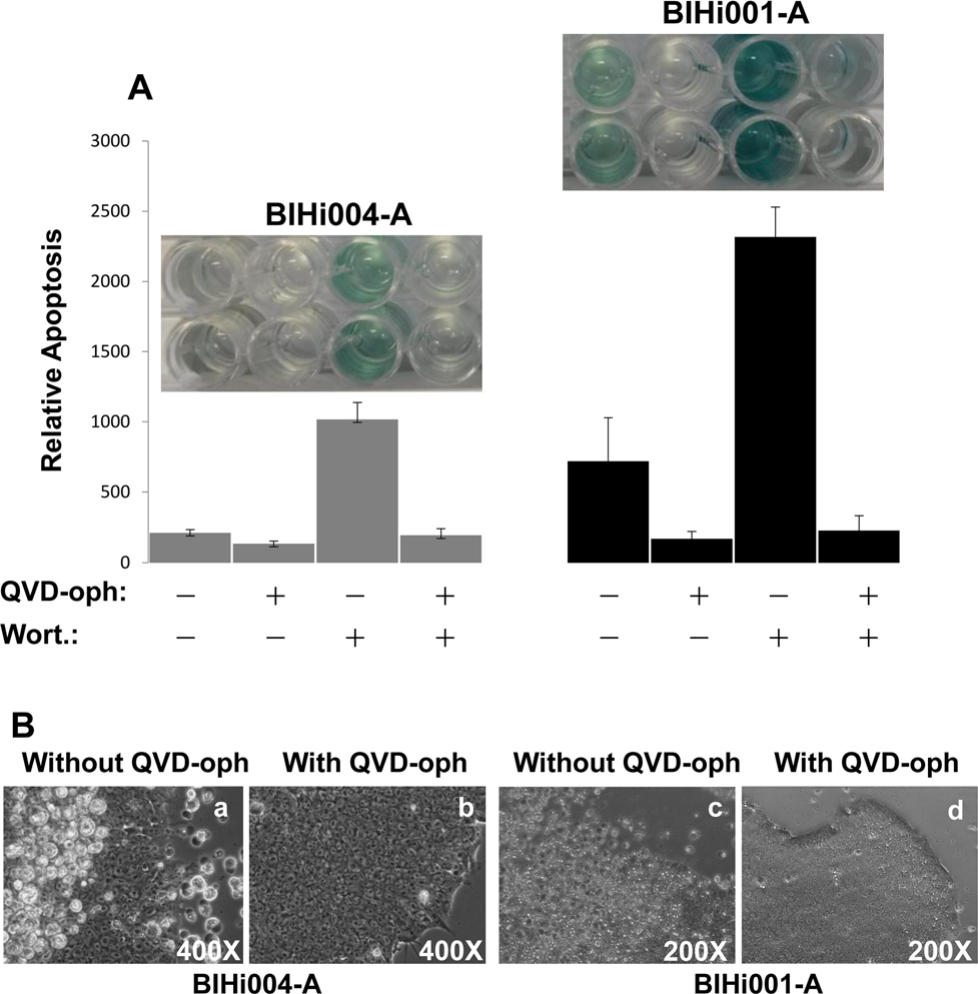
Wortmannin induced apoptosis in iPSCs is caspase dependent. (A) In support of our FACS analysis, apoptosis was quantified by determination of DNA fragmentation using ELISA cell death detection kit. The both iPSCs BIHi004-A and BIHi001-A were pretreated with QVD-oph or without QVD-oph and subsequently treated for additional 24h with 4μM wortmannin. Insets show picture of the ELISA with respect to color intensity. The color intensity is proportional to the level of apoptotic cells. (B) Pictures were taken 24 h after daily changing medium. (a,c) iPSCs as a control with a lot of dead cells in the supernatant after 24 h in two iPSCs in contrast to iPSCs pretreated with 10 μM caspase inhibitor (b,d).

The AD-iPSCs were cultured on 24 well plates coated with matrigel. Despite daily change of medium and quickly growing cultures, a significant number of cells died and detached, swimming in the medium both in coculture with feeder cells and under feeder-free conditions. In an own previous work [43], we found high basal apoptosis in all young and old iPSCs or even embryonic stem cell lines. Therefore we studied whether this abnormal cell death was caspase-dependent. AD-iPSCs were treated with 10 μM pan-caspase inhibitor (QVD-oph) and at the next day significantly fewer dead iPSCs with QVD-oph compared to untreated iPSCs were observed under light microscope (Fig 2A). Significant reduction of basal apoptosis was proven using FACS analysis (Fig 2B) and the cell death detection ELISA kit (Fig 2C). Cultivation with QVD-oph up to two weeks has barely shown morphological changes which could have marked differentiation. There were only marginal amounts of apoptotic cells with QVD-oph. In contrast, untreated AD-iPSCs exhibited many dead cells, as observed under the microscope. Furthermore using an alternative method to quantify apoptosis, BIHi004-A-iPSCs as well as BIHi001-A-iPSCs showed a significant increase of apoptosis induced by wortmannin, too. QVD-oph caused a decrease of wortmannin-induced apoptotic cells and also basal apoptotic cells compared to iPSCs without QVD-oph treatment (Fig 3A). Microscopic pictures of cells treated with QVD-oph and without are shown in Fig 3B (basal apoptosis).

### Morphological changes of iPSC colonies by wortmannin

**Fig 4:**
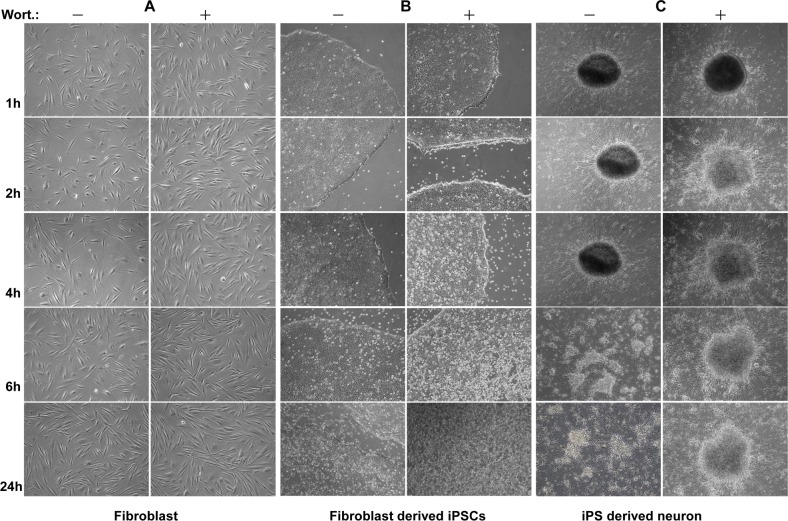
Strong apoptotic effect of wortmannin in iPSCs but not in parental fibroblasts and iPSC-derived neurons. Pictures of fibroblasts, fibroblast derived iPSC lines and iPSC-derived neurons were taken after treatment with 4 μM wortmannin at different time points (1 h, 2 h, 4 h, 6 h, 24 h) of fibroblasts, fibroblast deriveded iPSCs and iPSC-derived neurons. In contrast to both fibroblasts (NFH-46) (A) and iPSC-derived neurons (C), iPSCs (B) showed clear sensitivity to wortmannin induced apoptosis, resulting in a massive cell death with increasing time. The magnifications of all images are 40x.

As shown in Fig 4, striking morphological changes were observed in AD-iPSC colonies compared to original fibroblasts and iPSC-derived neurons. Phase contrast micrographs showed ragged cell edges after a short time of wortmannin treatment already at a lower concentration of the inhibitor versus non-treated cells with sharp edges. After a more prolonged incubation with wortmannin all iPSC colonies were detached. The detached cells swimming in medium exposed air balloon-like structures after 24 h (Fig 4B). In contrast, neither fibroblasts nor iPSC-derived neurons showed a visible morphological change like that, even after cell death (Fig 4A and 4C). Also after 48 h of wortmannin treatment no apoptosis could be observed in control fibroblasts and neurons (Fig 4A and 4C). Comparable results were obtained with BIHi004-A and BIHi001-A iPSCs (S2 Fig).

### Wortmannin-induced activation of the mitochondrial apoptotic pathway and formation of reactive oxygen species (ROS)

**Fig 5:**
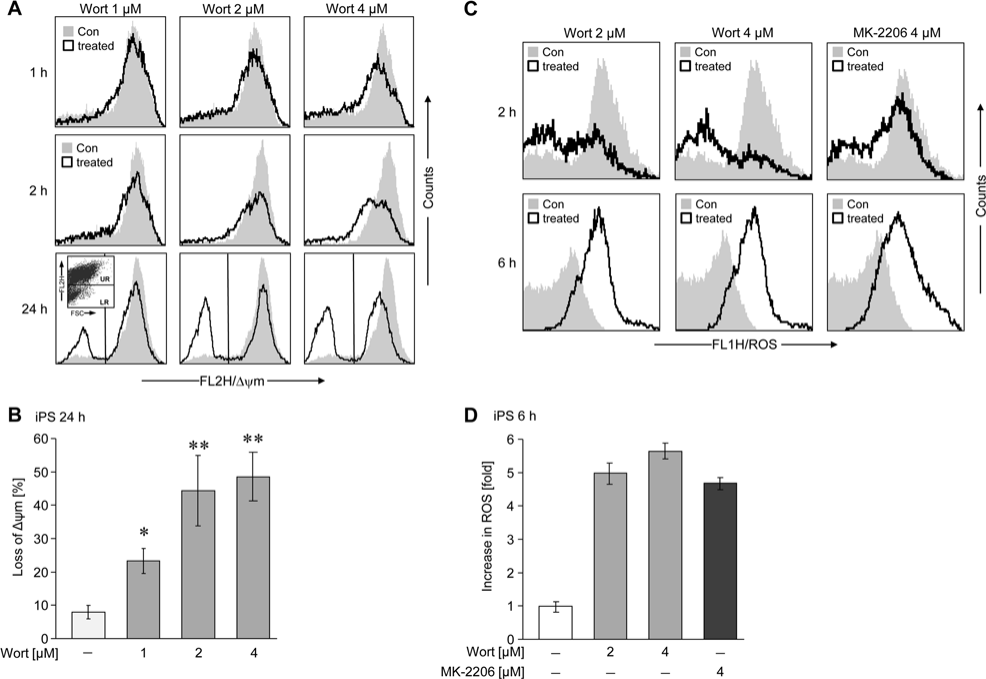
Decrease of membrane potential and increase of ROS production upon wortmannin treatment in AD-iPSCs. (A) Decreased membrane potential of mitochondria was determined by flow cytometry after TMRM^+^ staining in AD-iPSCs. Cells were treated with different concentrations of wortmannin (1 μM, 2 μM, 4 μM) for different times (1 h, 2 h, 24 h). Treated cells (open graphs) were compared to untreated controls (gray). (B) The quantitative data represent mean values of triplicate experiments +/-SD. (C) The production of ROS was determined after H2DCFDA staining in AD-iPSCs treated with two different concentrations of wortmannin (2–4 μM) or 4 μM of MK-2206 at two different time points (2h, 6h), by flow cytometry. Treated cells (open graphs) were compared to untreated controls (gray). (D) Quantitative data represent the median values of treated cells compared to control cells. ROS production was depicted as fold-increase, the control was set to 1. Two independent experiments with triplicates revealed comparable results

Treatment of AD-iPSCs with wortmannin already resulted at early time points (1 h) and at all concentrations tested (1–4 μM) in a decrease of the mitochondrial membrane potential, a common early feature of apoptosis, which further decreased with time (Fig 5A and 5B). This effect could be reproduced using AD-free clones BIHi004-A and BIHi001-A and different concentrations of 0.5–10 μM up to 24 h (S3 Fig). Collapse of mitochondrial membrane potential was dose- and time-dependent, while treatment for the longest time at highest concentration of wortmannin was most efficient. In the two AD-free iPS cell lines we could measure a decrease or no change of ROS production with different concentrations of wortmannin at various time points (S4A and S4B Fig). However, we could measure increasing ROS levels by wortmannin in AD-iPSCs (Fig 5C and 5D), but surprisingly α-tocopherol (Vitamin E) as ROS scavenger did not prevent wortmannin-induced apoptosis (S5 Fig). This could mean that ROS formation is just an accompanying effect, but not the responsible trigger of wortmannin-induced mechanism.

### Severe apoptosis induction by wortmannin in iPSCs, but not in neurons and fibroblasts

**Fig 6:**
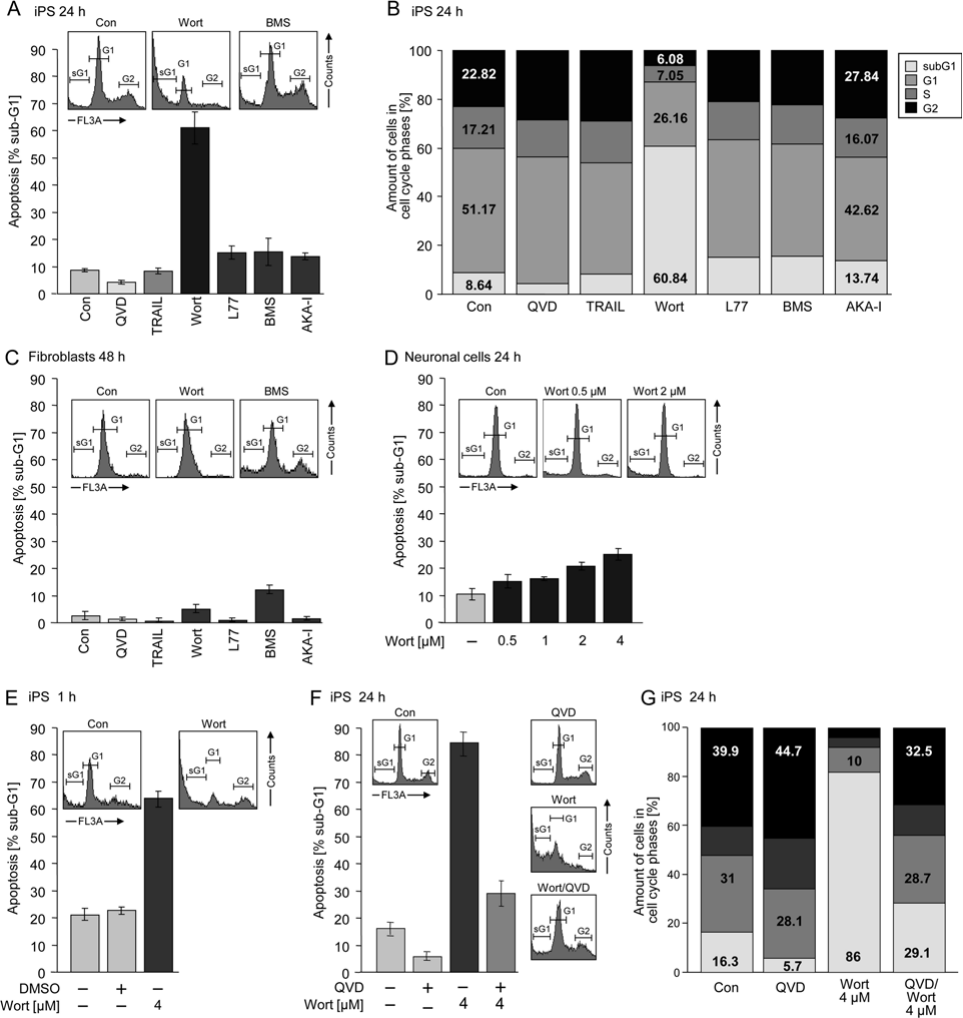
Wortmannin significantly induced apoptosis in AD-iPSCs. (A) Apoptosis (percentage of sub-G1 cells) was determined by cell cycle analysis in iPSCs treated for 24 h with 10 μM QVD-oph, 10ng/ml TRAIL, 4 μM wortmannin (Wort.), 4 μM L-779,450, 4 μM BMS, and 50 nM MLN-8237 (AKA-I). Insets: Histogram examples of cells treated with wortmannin or BMS as compared to controls (Con.). Sub-G1 cell populations are indicated (sG1). (B) Whole cell cycle analysis after treatment with small molecules mentioned in A with respect to the amount of different phases (sub-G1, G1, S, G2/M) in percent. (C) Apoptosis (percentage of sub-G1 cells) was determined by cell cycle analysis in orginal fibroblasts treated for 24 h with 10 μM QVD-oph, 10ng/ml TRAIL, 4 μM wortmannin, 4 μM L-779,450, 4 μM BMS, and 10 μM AKA-I. Insets: Histogram examples of cells treated with wortmannin or BMS compared to controls. Sub-G1 cell populations are indicated (sG1). (D) Apoptosis (percentage of sub-G1 cells) was determined by cell cycle analysis in iPSC-derived neurons treated with increasing concentrations (0.5, 1, 2, 4 μM) of wortmannin for 24 h. Insets: Histogram examples of cells treated with two different concentrations of wortmannin (0.5/2 μM) compared to controls. (E) Measurement of apoptosis after treatment with 4 μM wortmannin for 1 h compared to DMSO. (F) Apoptosis (percentage of sub-G1 cells) by 10 μM QVD-oph, 4 μM wortmannin or the combination of both in iPSCs after 24 h. (G) Percentages of different cell phases of iPSCs treated with 10 μM QVD-oph, 4 μM wortmannin or the combination of both for 24 h. Means and SDs are shown of three independent experiments in triplicates. Statistical significance (*; p < 0.05) is indicated for comparison of control cells and wortmannin-treated cells.

**Fig 7:**
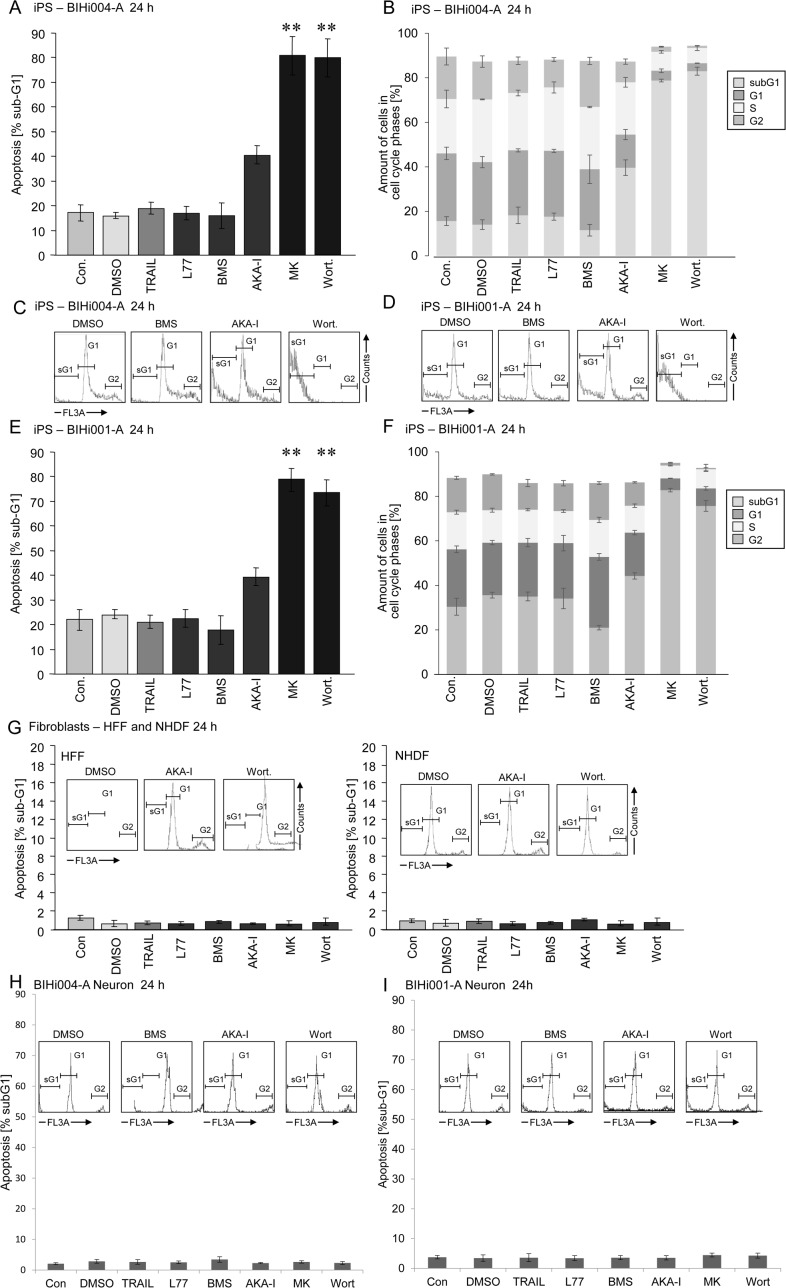
Wortmannin significantly induced apoptosis in BIHi001-A and BIHi004-A iPSC lines. (A,E) Apoptosis (percentage of sub-G1 cells) was determined by cell cycle analysis in iPSCs treated for 24 h with 10ng/ml TRAIL, 4 μM L-779,450, 4 μM BMS, 50 nM MLN-8237 (AKA-I), 4 μM MK-2206, 4 μM wortmannin (Wort.). (B,F) Whole cell cycle analysis after treatment with small molecules mentioned in A with respect to the amount of different phases (sub-G1, G1, S, G2/M) in percent. (C,D) Histogram examples of cells treated with BMS, AKA-I or wortmannin as compared to controls (DMSO). Sub-G1 cell populations are indicated (sG1). (G) Apoptosis (percentage of sub-G1 cells) was determined by cell cycle analysis in orginal fibroblasts (HFF and NHDF) treated for 24 h with 10ng/ml TRAIL, 4 μM L-779,450, 4 μM BMS, 50 nM MLN-8237 (AKA-I), 4 μM MK-2206, 4 μM wortmannin (Wort.). Insets: Histogram examples of cells treated with AKA-I or wortmannin compared to controls (DMSO). Sub-G1 cell populations are indicated (sG1). (H,I) Apoptosis (percentage of sub-G1 cells) was determined by cell cycle analysis in two iPSC-derived neurons treated for 24 h with 10ng/ml TRAIL, 4 μM L-779,450, 4 μM BMS, 50 nM MLN-8237 (AKA-I), 4 μM MK-2206, 4 μM wortmannin (Wort.). Insets: Histogram examples of cells treated with, BMS, AKA-I or wortmannin compared to controls (DMSO). Means and SDs are shown of three independent experiments in triplicates. Statistical significance (*; p < 0.05) is indicated for comparison of control cells and wortmannin-treated cells.

**Fig 8:**
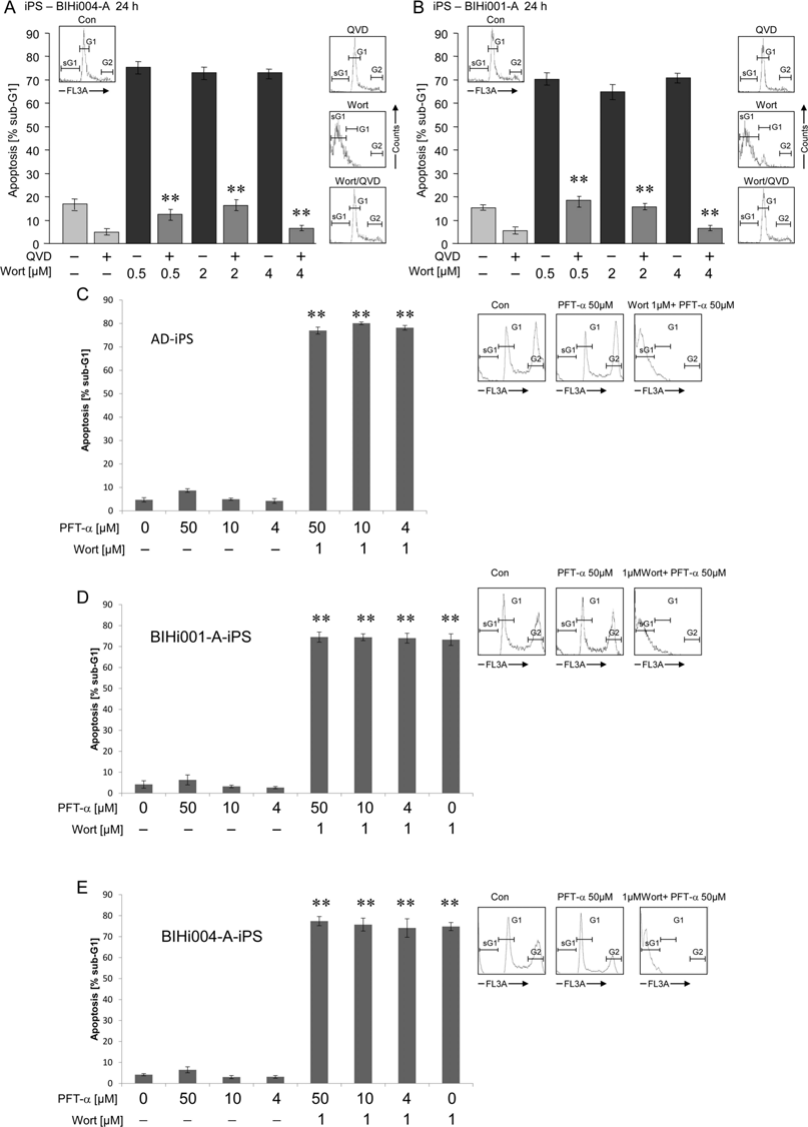
Wortmannin induced apoptosis can be blocked by pancaspase inhibitor but not by p53 inhibitor. (A,B) Apoptosis (percentage of sub-G1 cells) was determined by cell cycle analysis in both BHIi001-A and BHIi004-A iPSC lines treated with 10 μM pancaspasen Inhibitor (QVD-oph, 2h pretreatment) and subsequently with 4 μM wortmannin (Wort.) for additional 24h. Insets: Histogram examples of cells treated with wortmannin, QVD or QVD combined with Wortmannin as compared to controls (Con.). Sub-G1 cell populations are indicated (sG1). (C-E) Apoptosis (percentage of sub-G1 cells) was determined by cell cycle analysis in AD-iPSCs, BIHi001-A and BIHi004-A, all three iPSC lines pretreated with 4μM, 10μM and 50μM p53 inhibitor (PFT-alpha) and subsequently with 1μM wortmannin (Wort.) for additional 24h. Insets: Histogram examples of cells treated with PFT-alpha alone or PFT-alpha combined with wortmannin compared to controls. Sub-G1 cell populations are indicated (sG1). Means and SDs are shown of three independent experiments in triplicates. Statistical significance (*; p < 0.05) is indicated for comparison of control cells and wortmannin-treated cells.

Apoptotic cells of AD-iPSCs were clearly identified by cell cycle analysis as cells with fragmented DNA (sub-G1 cells, Fig 6A and 6C–6F, insets). Treatment with 4 μM of the PI3K inhibitor wortmannin for 24 h resulted in about 60% apoptosis in AD-iPSCs, whereas the B-RAF inhibitor L-779,450, the death ligand TRAIL, the IKK inhibitor BMS-345541 and the Aurora kinase A inhibitor MLN-8237 had, if any, marginal effects (Fig 6A and 6B). In original fibroblasts only BMS treatment resulted in a slight increase in apoptosis up to 12% (Fig 6C). Neuronal cells showed a concentration-dependent response to wortmannin after 24 h of treatment (Fig 6D). Induction of apoptosis by wortmannin in AD-iPSCs seemed to be an early event with 60% apoptosis after only 1 h treatment (Fig 6E). Treatment with the pan-caspase inhibitor QVD-oph reduced the basal apoptosis from 16% of untreated controls to 6% (Fig 6F). QVD-oph was also able to reduce wortmannin-induced apoptosis from 86% to 30%, leading to the presumption of a caspase-dependent apoptosis induction in iPSCs (Fig 6F). Simultaneously, treatment with QVD-oph resulted in an increased amount of cells at the G2 phase of growth (Fig 6G). Both AD-free iPSC lines were sensitive to apoptose induction by wortmannin as well as MK-2206 resulting in massive apoptosis in contrast to the other inhibitors used in this study. Surprisingly both clones were also a little sensitive to AKA-I inhibitor (Fig 7A–7F). The parental cells as well as iPS derived neurons were responsive to none of the inhibitors (Fig 7G–7I). Interestingly the use of pancaspase inhibitor QVD-oph in both AD-free iPSC lines showed a significant inhibition of basal apoptosis decreasing from 18% to 5% as well as wortmannin induced apoptosis (75% to 15%) (Fig 8A and 8B). But as also observed for AD-iPSCs, the detachment of the cells could not be prevented. In order to investigate the function of p53 in wortmannin induced apoptosis, we pretreated the cells with 4 μM, 10μM and 50μM PFT-alpha followed by 1μM wortmannin. In none of the iPSC lines used PFT-alpha was able to block the apoptotic effect of wortmannin. (Fig 8C–8E).

### Hoechst-33258 staining in iPSCs

**Fig 9:**
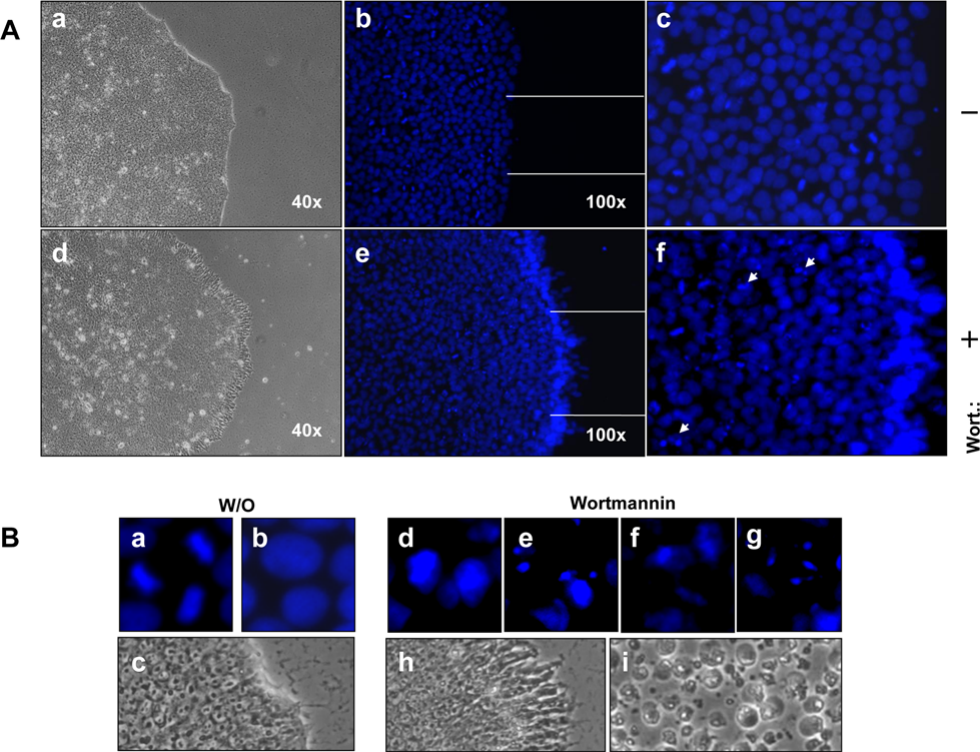
Wortmannin induced apoptosis in iPSCs causes nuclear condensation and fragmentation detected by Hoechst-33258 staining. (A) Phase contrast of untreated AD-iPSCs with sharp edges of round colonies (a) untreated AD-iPSC clones show mitotic cells, which can be seen by as intense blue (b-c). Phase contrast of wortmannin treated AD-iPSCs with frayed edges 1 h after treatment with 4μM wortmannin (d). A high proportion of cells showed clear indication of apoptosis by nuclear condensation and fragmentation, particularly pronounced at the edge of the colonies of AD-iPSCs (d-f) (examples indicated by arrows). B: Magnified images of untreated AD-iPSCs marked as mitotic cells (a), diffuse blue nucleus (b), sharp edge of untreated colony (c), magnified images of apoptotic cells with DNA condensation, DNA fragmentation (d-g). Changes in the morphology of the colonies one hour after treatment with wortmannin, and after 24 h complete detachment of the AD-iPSCs have been observed (h-i).

The prominent hallmarks of apoptosis are chromatin condensation and DNA fragmentation, which is a result of indirect activation of caspase-3. We visualised apoptosis, as a second proof, by Hoechst-33258 staining in the iPSCs 1 h after treatment mit 4 μm wortmannin. As shown in Fig 9 (AD-iPSCs) and S6 Fig. (BIHi004-A and BIHi001-A) wortmannin-treated iPSCs exhibited a brighter blue-whitish fluorescent appearance compared to untreated iPSCs. Furthermore, it was always striking that wortmannin treated iPSC clones clearly showed intense blue color at the edges. Interestingly, the mitotic cells in controls, but also in treated cells, were visible with deep intense blue staining, which suggested that the iPSCs were highly proliferative cells.

### Protein analysis of spontaneous apoptosis in AD-iPSCs

We reported in our previous work, that all young and old iPSCs and ESC lines used showed high apoptosis at the basal level. We could also show all iPSCs analyzed possessed a similar hypersensitivity to cytostatic agent-induced apoptosis in comparison to corresponding parental fibroblasts. This suggested that conversion of fibroblasts into iPSCs may reprogram the signaling apoptosis pathway on an epigenetic level.

**Fig 10:**
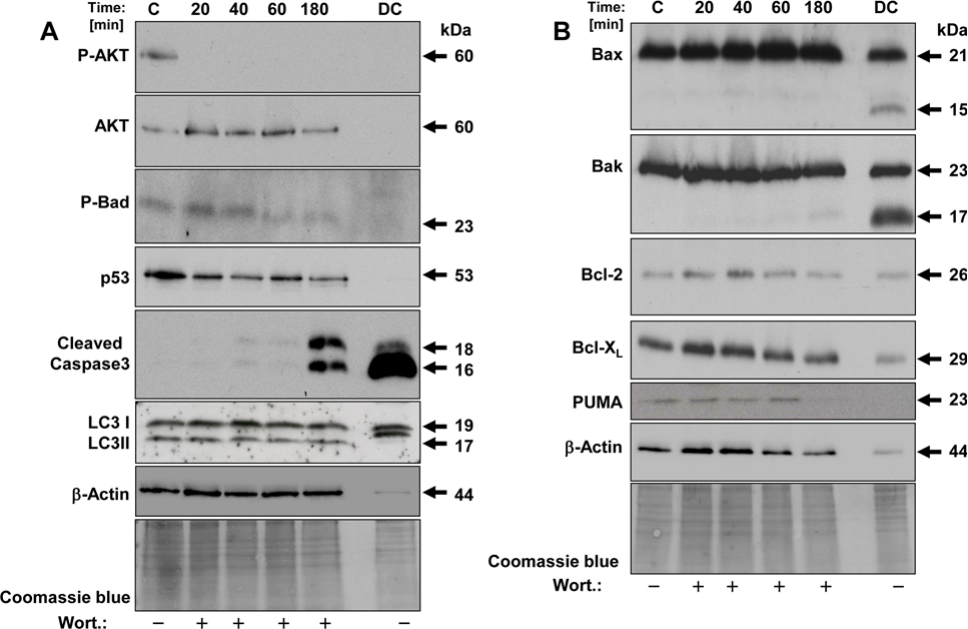
Western blot analysis of key proteins of apoptosis in wortmannin treated AD-iPSCs. Protein lysates were analyzed in AD-iPSCs after treatment with 4 μM wortmannin for different times. On the last lane of the blot at the right we subjected protein lysate of dead cells (DC) raised after 24 h in the supernatant to follow the basal apoptosis. Fourty μg of protein each were separated by SDS-PAGE (12%). Western blot analysis was used to monitor the expression of phosphorylated AKT (p-AKT, at serine 473) and total AKT, phosphorylated BAD and total BAD and p53. The anti-human Caspase-3 antibody used recognizes only the cleaved active form of caspase-3. The anti-human LC3A antibody recognizes both isomers, LC3I and LC3II. Furthermore we used anti-human BAX, BAK, BCL-2, BCL-X_L_ and PUMA antibodies, against members of the Bcl-2 family. Beta-actin and Coomassie blue staining were used to confirm similar protein loading across samples.

We have observed the same events in this study. Therefore we collected the dead cells (DC) and analysed apoptosis-related proteins as shown in Fig 10A and 10B in the last lane on the right side of the blots: The proteins P-AKT, AKT, P-BAD and p53 were not detectable (Fig 10A, lane DC). In contrast, prominent proapoptotic proteins such as BAX and BAK were strongly expressed and antiapoptotic proteins such as Bcl-X_L_ and Bcl-2 compared to proapoptotic proteins were only moderately expressed (Fig 10B, lane DC). In addition to specific bands of BAX and BAK we have detected additional bands, which are approximately 15 kDa and 17 kDa, repectively. Interestingly, we could detect a large amount of a distinct cleavage product of caspase-3, which indicates caspase-3 activation (Fig 10A, lane DC). Autophagy is a type of programmed cell death and it takes place with the help of autophagic marker proteins, like the cytoplasmic protein LC3-I and the membrane-binding LC3-II. Fig 10A, lane DC shows a shift of the LC3-II band, suggesting phosphorylation could have occurred here, which would have led to protein inactivation [44].

### Protein analysis of wortmannin induced apoptosis in AD-iPSCs

We analysed the expression of relevant proteins that are involved in classical apoptosis and PI3kinase pathways in AD-iPSCs in response to 4 μM wortmannin. After treatment times of 20, 40, 60 and 180 min., cells were lysed directly and the protein expression levels of p-AKT, AKT, p-BAD, p53, cleaved caspase-3, LC3I, LC3II, BAX, BAK, BCL-2, BCL-X_L_ and PUMA were investigated (Fig 10A and 10B). Expression of AKT, a downstream target protein in the PI3K pathway, remained unchanged, whereas the phosphorylation of AKT as survival signal disappeared after 20 min. Pro-apoptotic activity of BAD is regulated through its phosphorylation via phosphorylated AKT. In our analysis we detected a decrease of phosphorylated BAD indicating pro-apototic activation. Furthermore, we did not observe any changes in LC3I and LC3II as autophagy markers, but a time-dependent decrease in expression of p53. According to these results and to the lacking effect of PFT-alpha (Fig 8C–8E), it seems likely that p53 protein is not involved in wortmannin induced apoptosis in our study. However, wortmannin-induced apoptosis was caspase-dependent, since we found a significant increase in the cleaved form of caspase-3 (Fig 10A).

### The role of BCL-2 proteins in wortmannin-induced apoptosis in AD-iPSCs

The ratio of anti-apoptotic to pro-apoptotic Bcl-2 family members determines the cell fate [17]. Protein analysis showed equal amounts of BAX and BAK as pro-apoptotic and Bcl-2 and Bcl-X_L_ as anti-apoptotic proteins. But the ratio of pro-apoptotic to anti-apoptotic proteins was already high in the control AD-iPSCs. PUMA, another pro-apoptotic protein, regulated via p53, remained unchanged until 60 min. and was thereafter not detectable (Fig 10B).

### Altered basal protein expression after differentiation of AD-iPSCs into neurons

**Fig 11:**
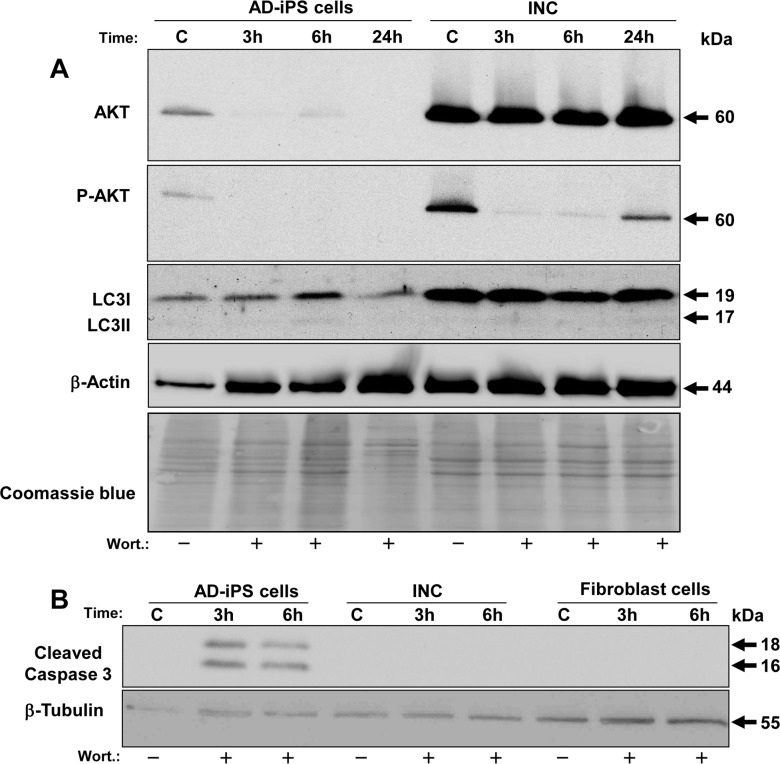
Comparative analysis of proteins between AD-iPSCs and AD-iPSC-derived neurons after wortmannin treatment. Protein lysates of AD-iPSCs and iPSC-derived neurons (INC) were analyzed after treatment with 4μM wortmannin for different times. Fourty μg of protein each were separated by SDS-PAGE (12%). (A) Western blot analysis was used to monitor the expression of total and phosphorylated Akt (p-Akt, at serine 473). The anti-human LC3A recognizes both isoforms LC3I and LC3II and could also show phosphorylated LC3II. (B) iPSCs compared to iPSC-derived neurons and original fibroblasts. The anti-human Caspase-3 used recognizes only the cleaved active form of caspase-3. Beta-tubulin and Coomassie blue staining were used to confirm similar protein loading across samples.

Interestingly, differential protein expression between iPSCs and iPSC-derived neurons with respect to AKT, p-AKT and LC3 proteins could be detected. As shown in Fig 11A the basal expression of AKT in AD-iPSC-derived neurons was tenfold higher compared to AD-iPSCs. Furthermore, higher levels of phosphorylated AKT were detected in neurons. On the other hand, p-AKT was significantly downregulated (8-fold) in neurons after 3 and 6 h of wortmannin treatment, and 24 h later the cells upregulated p-AKT again and were alive. The basal expression of LC3 was 5-fold higher than in AD-iPSCs. Regarding caspase-3 activity, we detected the cleaved caspase-3 only in AD-iPSCs after 3 h of wortmannin treatment, but not in AD-iPSC-derived neurons or orginal fibroblasts, as shown in Fig 11B.

## Discussion

A sensational development in the field of regenerative medicine and stem cell research was the achievement of cells with many properties in common with hESCs without the use of embryos [1,45]. In order to study disease mechanisms in a dish, the iPSC technology gives us valuable insight into poorly accessible disease-affected materials such as brain cells and has several advantages over the current possibilities of investigation. Meanwhile several researchers worldwide have succeeded to develop patient-specific iPSCs from various diseased somatic cells and to differentiate them into desired cell types that are defect or lost in certain diseases [46]. Nevertheless there are several obstacles that must be resolved before routine use. The system of gene delivery will have to be improved in order to avoid genetic destabilization, the method should become faster and more efficient, and differentiated cells will have to be free of “contaminating” iPSCs after differentiation in order to avoid tumor formation in case of cell transplantation. Since they may recapitulate a similar genetic background, they may only be used for cell transplantation after correction of the causes of the disease. Unlike human ESCs, human iPSCs acquire most chromosomal aberrations during induction of cellular reprogramming or long-term cultivation, which may cause tumorigenity and apoptosis resistance [47,48]. Chromosomal aberration is one of the important reasons for tumorigenicity in cancer cells. In a previous study we have reported that the generated iPSCs acquired chromosomal aberrations [43]. This is in accordance to other iPSC studies [49]. Surprisingly, despite their continuous growth under optimal culture conditions or/and chromosomal aberrations, all developed iPSCs and ESCs displayed high spontaneous apoptosis. They also were highly sensitive to apoptosis inducers such as cytostatic actinomycine C [43] and in another report the iPSCs were highly sensitive for apoptosis after irradiation treatment, [50]. Nevertheless the same iPSCs used in our study or in the other study formed teratoma in mice [5,43,51,52]. Therefore, the tumour-like iPSCs would be very interesting as a model to study tumorigenesis, since they behave as cancer cells due to unlimited proliferation, formation of teratoma in mice and genetic aberrations.

On the other hand, two studies dealed with the mechanism of cell death in iPSCs and ESCs using small molecules. These were directed to pathways involved in cell survival, apoptosis and adhesion molecule signaling and tried to elucidate the underlying mechanisms and to achieve an improvement in keeping "well-doing" iPSCs during passages and culture periods with reduced basal apoptosis [53,54]. Addition of Rock inhibitor to extracellular matrix was shown to significantly reduce spontaneous basal cell death and to increase plating efficiency of hESC and hiPSCs during a culture period. The proposed mechanisms for this positive effect were the induced expression of integrins αv, α6, ß1, and ß5 detected by RT-qPCR analysis [53]. Furthermore and regarding apoptosis, the use of Maxadilan activated the pituitary adenylate cyclase-activating polypeptide (PACAP) type I receptor (PAC1) in iPSCs, prevented apoptosis induced by ultraviolet C (UVC) and interestingly had no effect on pluripotent properties and karyotype state [54]. The anti-apoptotic effect was suggested to be due to downregulation of caspase-3 and caspase-8. We have also observed a conspicuous spontaneous basal cell death under both conditions, on feeder cells and in feeder-free medium in our study. Therefore, we collected the dead cells from medium and analyzed their proteins in comparison to adherent AD-iPSCs in terms of prominent apoptosis-associated proteins. The Western blot analysis of dead AD-iPSCs revealed a significantly high expression of the pro-apoptotic proteins BAX and BAK compared to the anti-apoptotic proteins BCL-2 and BCL-X_L_, which control the release of pro-apoptotic proteins, such as cytochrome C, from mitochondria into the cytosol [55]. The relative concentration of pro- and anti-apoptotic proteins may act as a rheostat for cell suicide programs and lead to a sensitization for basal apoptosis [17]. We have also detected a shift of the lower band of marker of autophagy LC3II protein compared to adherent AD-iPSCs, which is probably due to the phosphorylation and subsequent inactivation of this protein [44]. Furthermore, we detected a significant increase of cleaved caspase-3 compared with adherent AD-iPSCs as control. Consistent with our findings of enhanced caspase activity, the use of the pan-caspase inhibitor QVD-oph clearly reduced the number of dead AD-iPSCs under constant morphology and growth up to two weeks. We conclude that caspase-3 is an essential effector caspase involved in the spontaneous death of iPSCs under feeder and feeder free based matrigel-coated culture conditions. However, we cannot exclude that other caspases of this family play a role in this context. Therefore it would be interesting to apply selective caspase inhibitors for caspase-3, -8 and -9, in order to check their role regading spontaneous death in this cell model.

The other important finding in this study is that wortmannin, as an inhibitor of the PI3K signaling pathway, dramatically induced iPSC apoptosis in a caspase-dependent manner as shown not only for AD-iPSCs, but also for two AD-free iPSC lines. The PI3K signaling pathway and especially its downstream protein AKT are of great importance in mouse ESCs and early embryos and are considered crucial for unlimited proliferation and survival as well as for inhibition of the apoptotic processes [21]. AKT can phosphorylate and dephosphorylate various target proteins to ensure the healthy normal growth and viability of the cells. Overexpression of AKT in this pathway due to mutations can contribute to cell tumorigenesis. It can cause apoptosis resistance and aggressive growth behavior, e.g. by dysregulation of Bad and p53 which are target genes of AKT [56]. It has been recently reported that melanoma cells are resistant to wortmannin, but on the other hand they become sensitized to apoptosis in combination with TRAIL [26]. This is due to formation of ROS which leads to BAX activation by its phosphorylation and downregulation of phosphorylated AKT [26]. We also detected an increase of ROS and markedly decreased P-AKT in our AD-iPSCs after wortmannin treatment, although ROS increase could not be seen in the other two iPSC lines used. But ROS levels can be extremely time-dependent so that an increase in the BIHi-clones cannot be excluded. High expression of AKT or a deregulated PI3K-AKT signaling pathway was responsible for the resistance of MCF-7 to various chemotherapeutics such as etoposide, doxorubicin and camptothecin. However, application of wortmannin in breast cancer cells MCF-7 induced apoptosis with pronounced morphological and biochemical apoptotic properties, suggesting AKT as a potential target gene in breast cancer cell therapy [57]. In iPSCs, however, the role of the PI3K/AKT pathway has been, to our knowledge, not yet defined.

For this work we have reviewed a number of small biological molecules in terms of their effectiveness to cell viability and apoptosis in iPSCs, but wortmannin and MK-2206 were the only to induce cell death in iPSCs, and that with a dramatic effect. Surprisingly, orginal fibroblasts as well as iPSC-derived neurons showed no response to wortmannin regarding apoptosis induction, although AKT protein levels were significantly high in contrast to AD-iPSCs. But high expression of AKT could perhaps especially be the reason for the lack of response, which was also described for MCF-7 and other tumor cells resistant to apoptosis and chemotherapeutics as mentioned above [57]. Different AKT protein levels from original fibroblasts, AD-iPSCs and AD-iPSC-derived neurons with the same genetic origin could be due to epigenetic changes by reprogramming as well as by differentiation. However, there are several recent studies showing that iPSCs retain the genetic and epigenetic background of the cells of origin [58–60]. On the other hand, it is controversially reported that iPSCs and ESCs are not close to each other in terms of molecular nature, while others indicate great similarities in this respect [59,61,62]. In contrast to iPSCs, the original fibroblasts used here showed no apoptotic effect for wortmannin treatment even after 48 h, but instead NHF-46 were more sensitive to the IKK inhibitor BMS-345541 affecting the NF-kappaB signaling pathway so that AD-iPSCs seemed to have acquired resistance to the latter inhibitor. Since NF-kappaB signaling has been described as very important for maintaining the undifferentiated state of iPSCs [63], high activity of the pathway as assumed above for the PI3K/AKT pathway could be the cause of the lack of reaction. For confirmation, we also treated another series of skin fibroblasts obtained from a young healthy woman under identical conditions, but apoptosis induction was not seen upon wortmannin treatment (S7 Fig).

In the iPSCs we could significantly inhibit wortmannin-induced apoptosis (with involvement of caspase-3) by 50–80% using the pan-caspase inhibitor QVD-oph. On the other hand, the caspase inhibitor could not prevent detachment of iPSCs. It is, therefore, assumed that wortmannin possibly exerts its effect via multiple pathways. We subsequently focused on the role of p53 activation in mediating apoptotic response by wortmannin using PFT-alpha in all iPSCs, but we could not detect any inhibiton of apoptosis. Wortmannin did not change the ratio of the pro- and anti-apoptotic Bcl-2 family members BAX, BAK to BCL-2, BCL-X_L_ in AD-iPSCs, which remained at its high level. Several reports demonstrated that the ratio of pro-apoptotic to anti-apoptotic Bcl-2 proteins determines cell fate in cancer cells, whether cells can stay alive or die after treatment with apoptotic stimuli [17,64]. Based on our findings and other studies, we have hypothesized that the iPSCs should be sensitive to TRAIL treatment, but we did not detect apoptosis on a morphological or biochemical level. Probably the TRAIL-induced apoptosis signaling pathway is not sufficiently developed in our iPSCs and they could behave like tumor cells with resistance to apoptosis. It would be interesting to check further anti-apoptotic proteins such as inhibitors of apoptosis (IAPs) and others. Further protein analysis revealed that after short incubation times with wortmannin phosphorylated AKT began to decrease with increasing time in AD-iPSCs, underlined by consistent AKT expression. Phosphorylation of AKT provides survival and blocking of apoptosis. Interestingly, AD-iPSC-derived neurons behaved similarly (on a higher initial level), but after 24 hours P-AKT increased again and we observed no relevant apoptosis induction after treatment with wortmannin.

In conclusion, our results indicate the importance of the PI3K-AKT signaling pathway and of caspase-3 activity in our AD-iPSCs derived from skin fibroblasts, concerning survival and apoptosis. As far as we know, we were the first to efficiently reduce basal cell death using QVD-oph, to avoid spontaneous differentiation over two weeks and to highlight the role of the PI3K pathway for (AD-derived) iPSCs. The specificity of action could also be shown with low levels of wortmannin. Different responses and expression levels of the three cell types, the original cells, AD-iPSCs and AD-iPSC-derived neuronal cells suggest changes in the epigenetic background. Regarding iPSC-based therapies with the risk of tumour development, apoptotic elimination of remaining undifferentiated iPSCs using wortmannin could be a suitable solution for tumour prevention. Therefore, it would be interesting to investigate the apoptotic effect of wortmannin in vivo after injection of iPSCs in mice. Interestingly, the Alzheimer and Alzheimer-free iPSC lines showed no differences in apoptotic behaviour mediated by wortmannin treatment. Since the iPSCs research is growing rapidly and gets enormous importance for the elucidation of the molecular pathogenesis and on the other side valuable insight for stem cell-based therapy, we hope that these findings are helpful for understanding the mechanisms of survival and apoptosis.

## Supporting Information

S1 Fig Characterization of iPSC colonies for pluripotency and neuronal differentiation. A) RT-qPCR analysis of pluripotency gene expression of OCT3/4, Sox-2, Nanog in both iPSC lines, BHIi004-A and BHIi001-A. WAe001-A (H1) embryonic stell cells and HFF were used here as positive and negative controls for pluripotency genes, respectively. B) (a-c): They were positive for pluripotency-associated alkaline phosphatase (AP) staining in a feeder-free system. (d-f) Typical iPSC morphology. Neuronal differentiation of both iPSCs after infection of lentiviral vector containing Ngn2 (h, light picture) and Ngn2 with GFP as reporter gene (g, green fluorescent protein).
(PDF)

S2 Fig Strong apoptotic effect of wortmannin in both BIHi001-A and BIHi004-A iPSC lines but not in their parental fibroblasts and iPSC-derived neurons. Pictures were taken after treatment with 4 μM wortmannin at different times (1 h, 2 h, 6 h, 24 h) of two fibroblast cell lines, corresponding fibroblast derived iPSCs and iPSC-derived neurons. In contrast to both fibroblast cell lines (HFF and NHDF) (A) and iPSC-derived neurons (C), iPSCs BHIi001-A and BHIi004-A (B) showed clear sensitivity to wortmannin induced apoptosis, resulting in a massive cell death with increasing time. The magnifications of all images are 40x.
(PDF)

S3 Fig Decrease of membrane potential upon wortmannin treatment in BIHi001-A and BIHi004-A iPSC lines. (A,C) Decreased membrane potential of mitochondria was determined by flow cytometry after TMRM^+^ staining in iPSCs BIHi001-A and BIHi004-A. Cells were treated with different concentrations of wortmannin (0.5 μM, 2 μM, 4 μM, 10μM) for different times (2 h, 3 h, 7h, 24 h). Treated cells (red) were compared to untreated controls (gray). (B,D) The quantitative data represent mean values of triplicate experiments +/-SD.
(PDF)

S4 Fig No changes in ROS production upon wortmannin treatment in both iPSCs BIHi001-A and BIHi004-A. (A,B) The production of ROS was determined after H2DCFDA staining in AD-iPSCs treated with three different concentrations of wortmannin (0,5–4 μM) or at three different time points (0.5h, 2h, 4h) by flow cytometry. Treated cells (open graphs) were compared to untreated controls (pink). Two independent experiments with triplicates of both iPS cell lines revealed comparable results.
(PDF)

S5 Fig Wortmannin induced apoptosis was not blocked applying Vitamin E (Vit E) in AD- iPSCs. Apoptosis (percentage of sub-G1 cells) was determined by cell cycle analysis in AD-iPSCs pretreated for 2 h with 10 μM alpha-tocopherol (Vit E) and subsequently treated for 24h with 2μM and 4μM wortmannin. Histogram examples of cells treated with wortmannin alone or in combination with Vit E as compared to controls (Con.). Sub-G1 cell populations are indicated (sG1).
(PDF)

S6 Fig Wortmannin induced apoptosis in iPSCs causes nuclear condensation and fragmentation detected by Hoechst-33258 staining. (A) Phase contrast of untreated iPSCs BIHi001-A and BIHi004-A with sharp edges of round colonies (a,b). Untreated iPSC clones show mitotic cells, which can be seen as intense staining, but most of the cells are diffuse blue (e,f). Wortmannin treated iPSCs with frayed edges 2 h after treatment with 4μM wortmannin can be seen in phase contrast (c,d) and as intense blue (g,h). A high proportion of cells showed clear indication of apoptosis by nuclear condensation and fragmentation, particularly pronounced at the edge of the colonies of iPSCs).
(PDF)

S7 Fig No apoptotic effect of wortmannin in healthy fibroblasts. (A) Apoptosis (percentage of sub-G1 cells) was determined by cell cycle analysis in healthy fibroblasts treated for 48h h with 4 μM wortmannin, compared to controls. Sub-G1 cell populations are indicated (sG1). (B) Pictures of fibroblasts were taken after 48 h treatment with 4 μM wortmannin. Magnification 40x.
(PDF)

S1 Table List of primer pairs for pluripotency genes used in this study. (XLSX)

## Abbreviations

PI3K: Phosphoinositid-3-Kinase
ESCs: embryonic stem cells
iPSCs: induced pluripotent stem cells
AKT: protein kinase B
