# Supplementary figures and images for: PI3K/AKT Signaling Pathway Is Essential for Survival of Induced Pluripotent Stem Cells
Source: PLoS One. 2016 May 3;11(5):e0154770. doi: 10.1371/journal.pone.0154770 (PMC4854383; doi:10.1371/journal.pone.0154770)

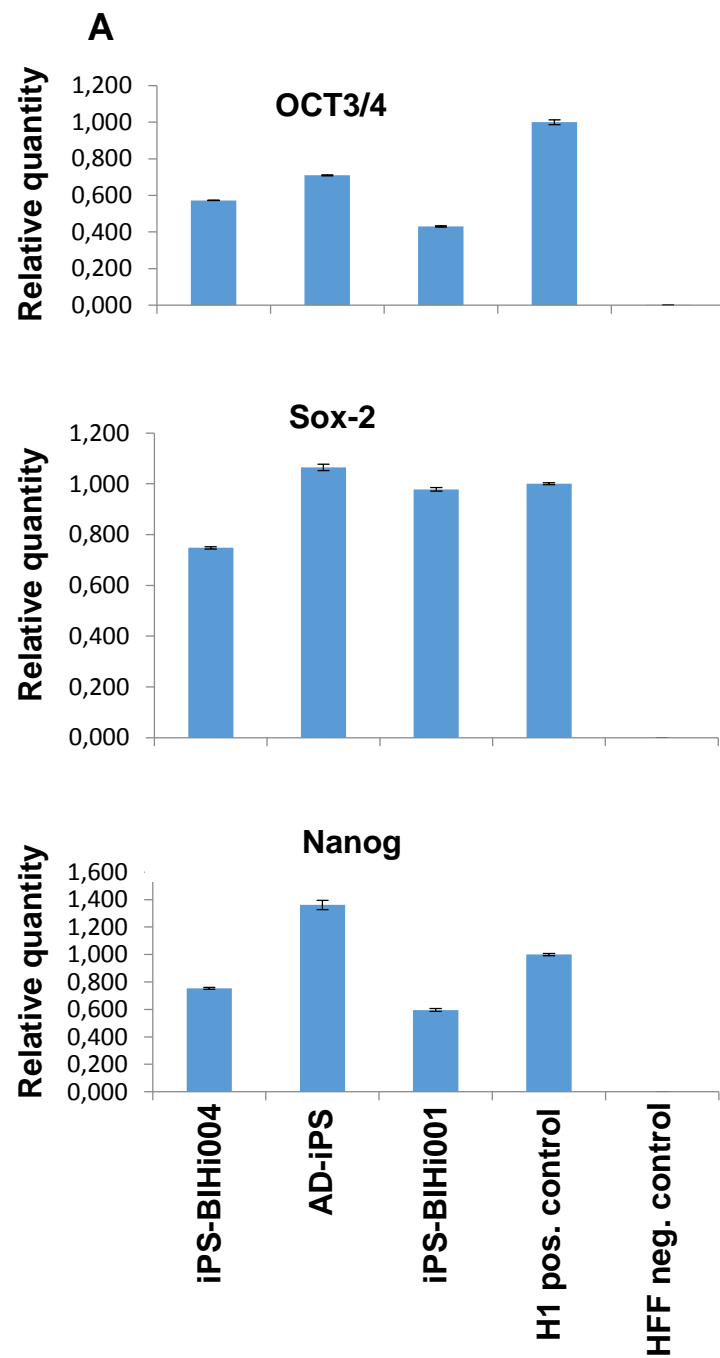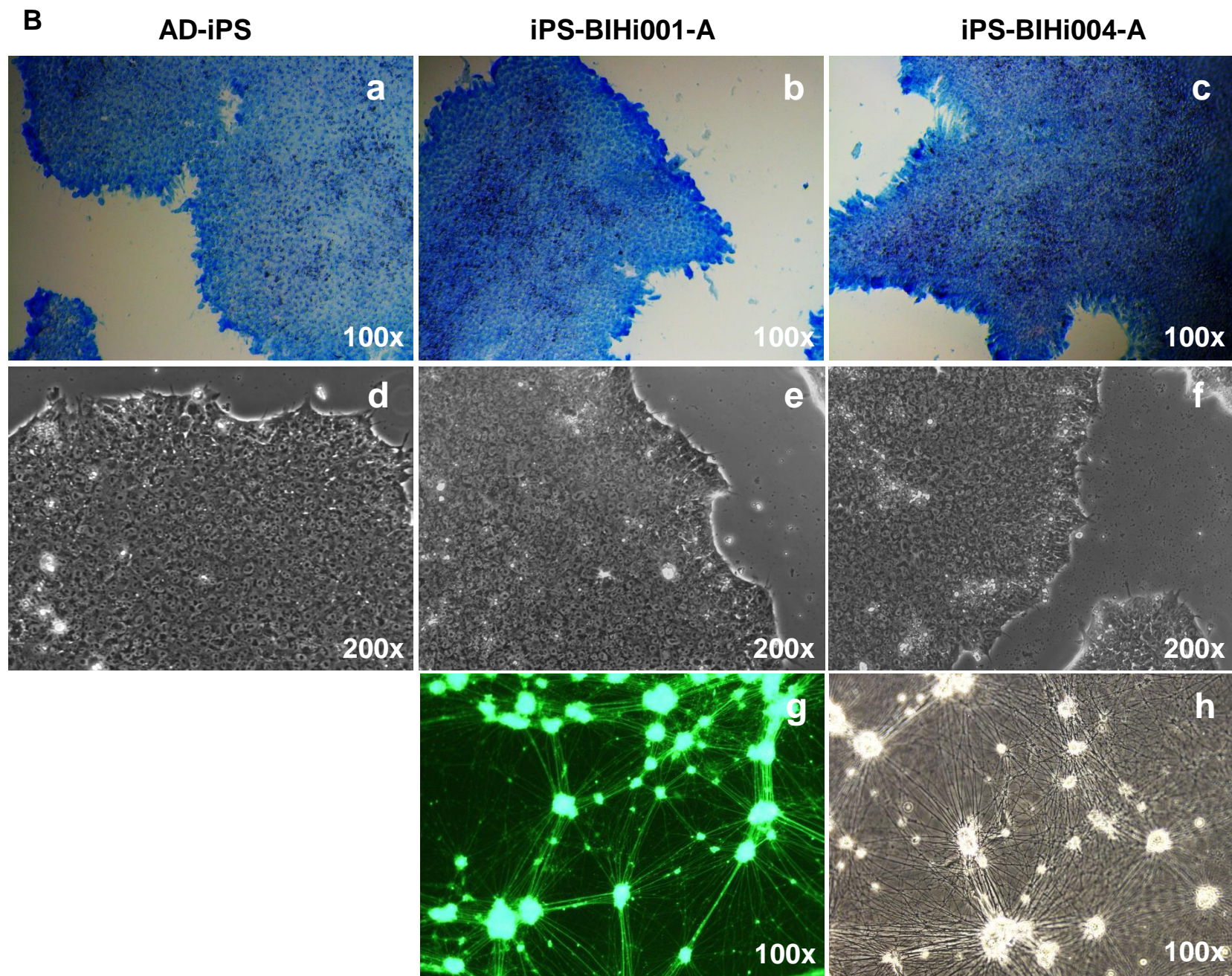

Supplement: S1 Fig — A) RT-qPCR analysis of pluripotency gene expression of OCT3/4, Sox-2, Nanog in both iPSC lines, BHIi004-A and BHIi001-A. WAe001-A (H1) embryonic stell cells and HFF were used here as positive and negative controls for pluripotency genes, respectively. B) (a-c): They were positive for pluripotency-associated alkaline phosphatase (AP) staining in a feeder-free system. (d-f) Typical iPSC morphology. Neuronal differentiation of both iPSCs after infection of lentiviral vector containing Ngn2 (h, light picture) and Ngn2 with GFP as reporter gene (g, green fluorescent protein). (PDF) [file pone.0154770.s001.pdf]

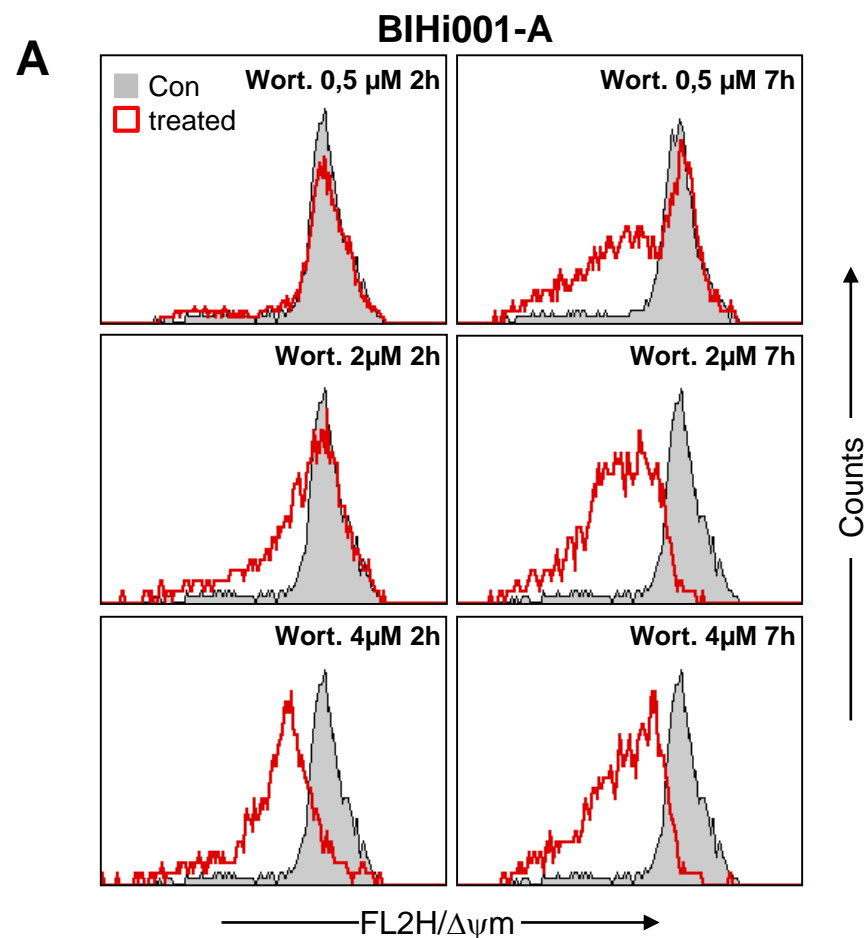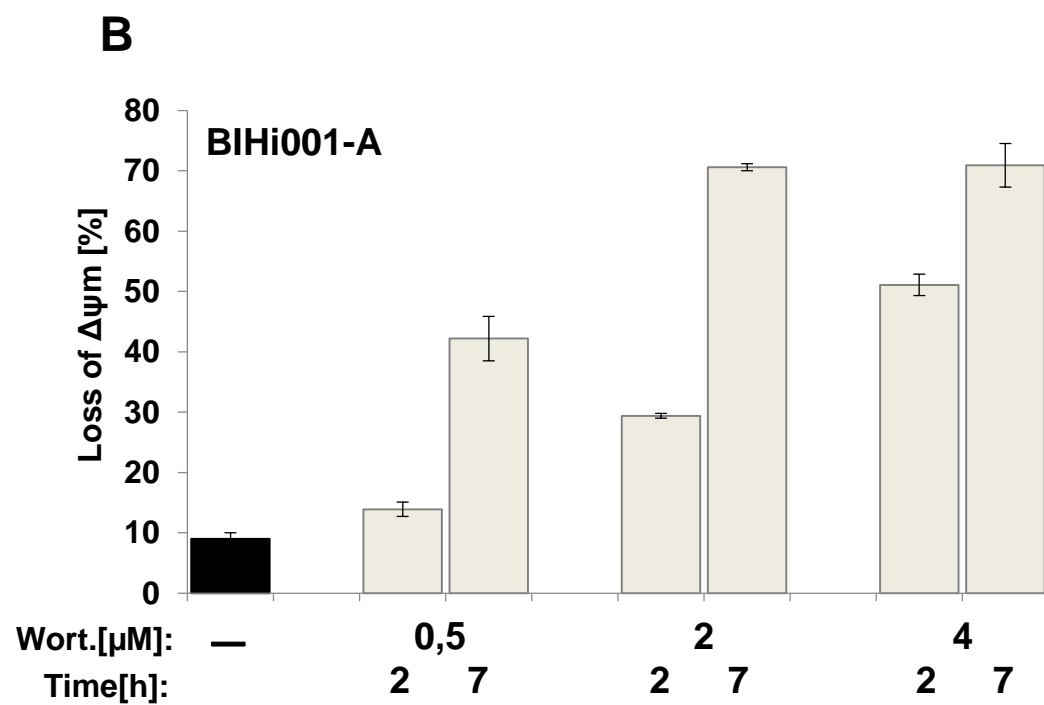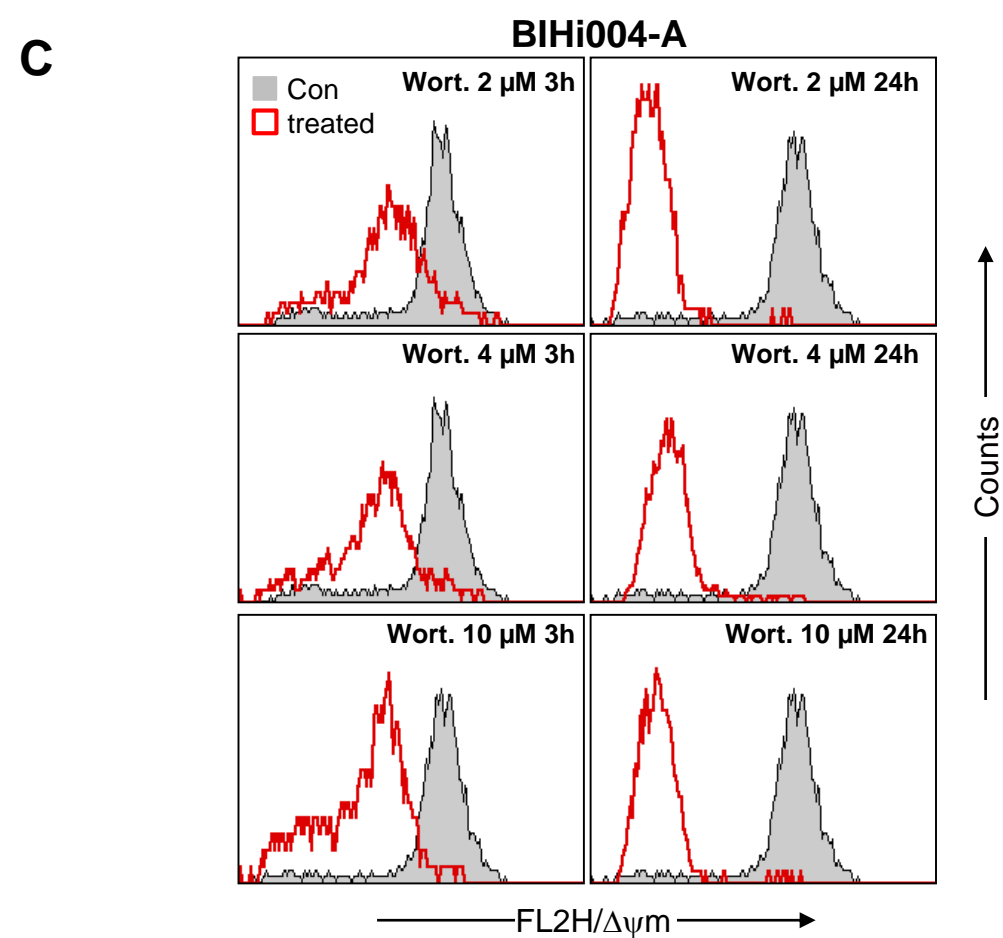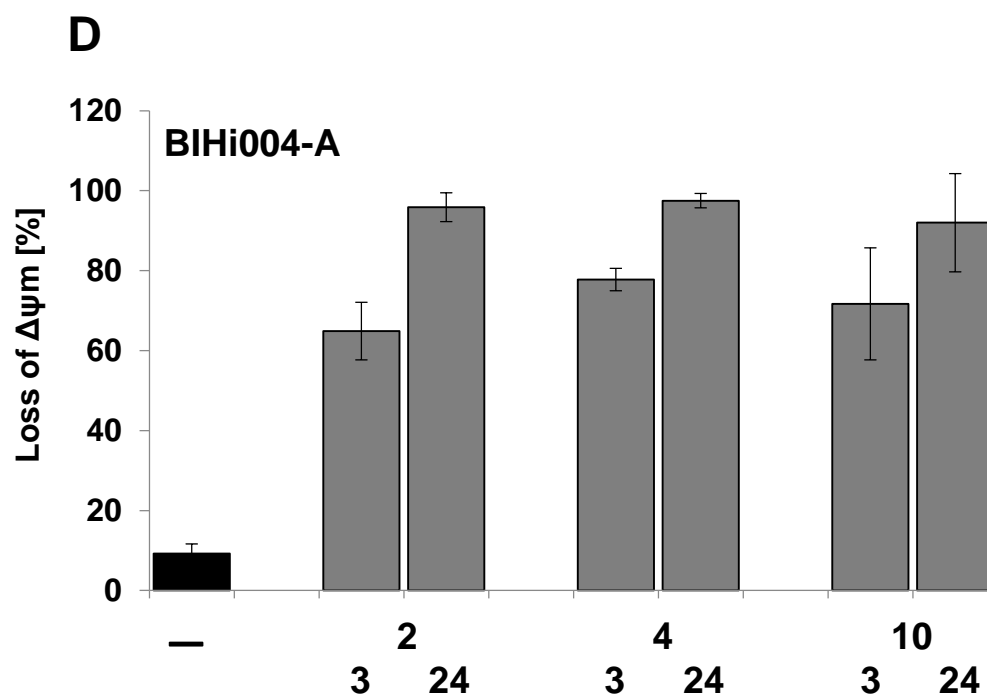

Supplement: S3 Fig — (A,C) Decreased membrane potential of mitochondria was determined by flow cytometry after TMRM+ staining in iPSCs BIHi001-A and BIHi004-A. Cells were treated with different concentrations of wortmannin (0.5 μM, 2 μM, 4 μM, 10μM) for different times (2 h, 3 h, 7h, 24 h). Treated cells (red) were compared to untreated controls (gray). (B,D) The quantitative data represent mean values of triplicate experiments +/-SD. (PDF) [file pone.0154770.s003.pdf]

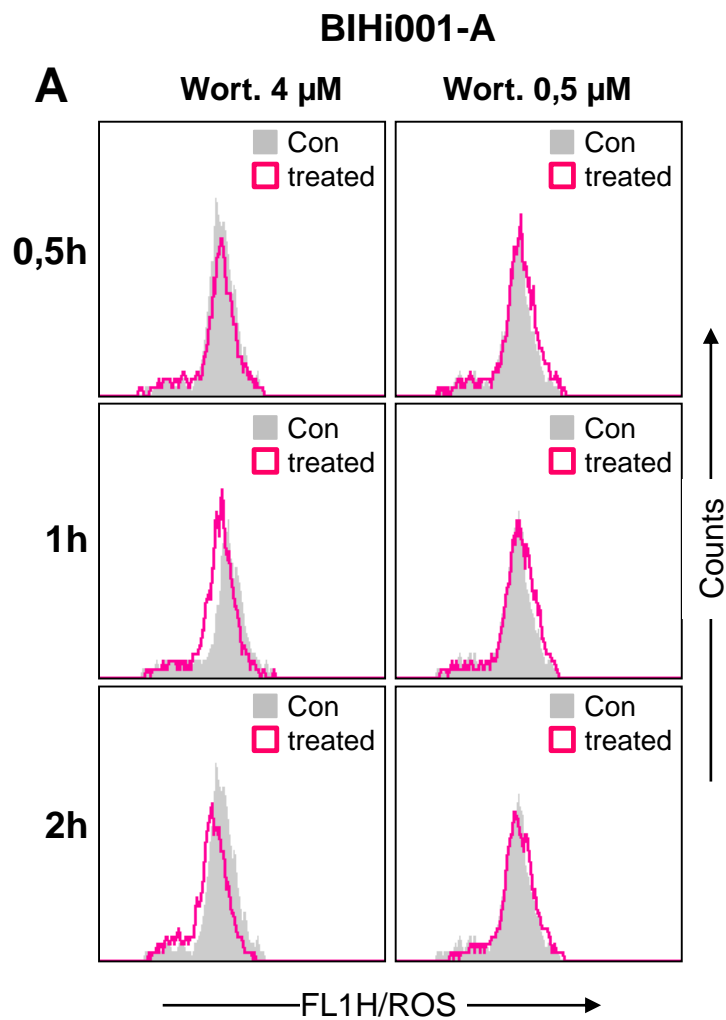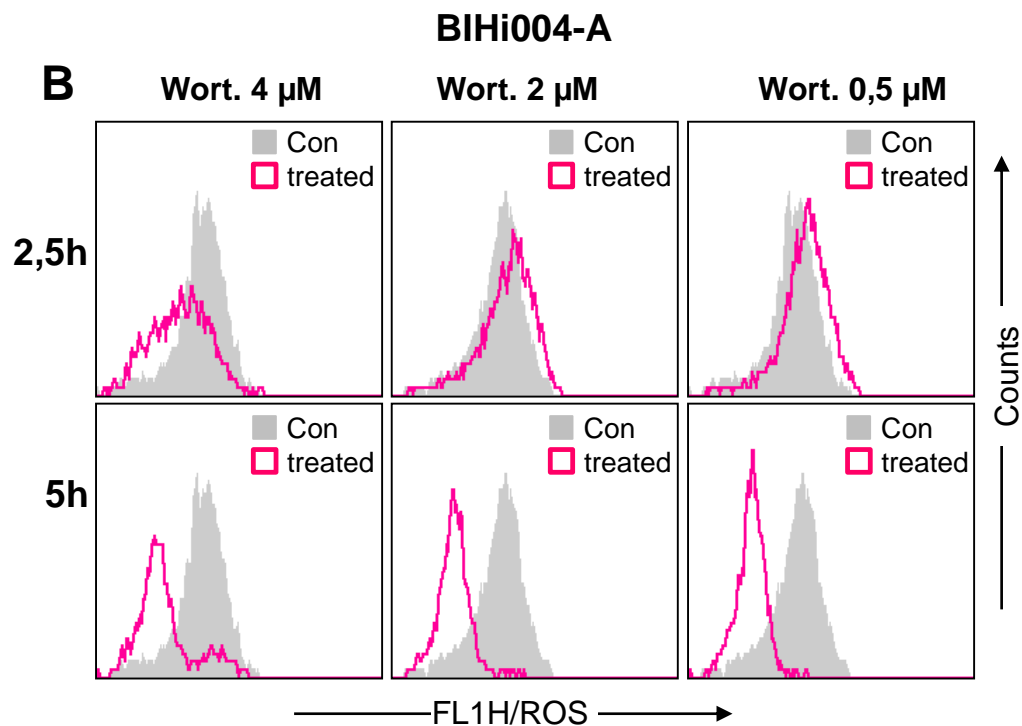

Supplement: S4 Fig — (A,B) The production of ROS was determined after H2DCFDA staining in AD-iPSCs treated with three different concentrations of wortmannin (0,5–4 μM) or at three different time points (0.5h, 2h, 4h) by flow cytometry. Treated cells (open graphs) were compared to untreated controls (pink). Two independent experiments with triplicates of both iPS cell lines revealed comparable results. (PDF) [file pone.0154770.s004.pdf]

**AD-iPSCs**  
**Incubation time : 24h**

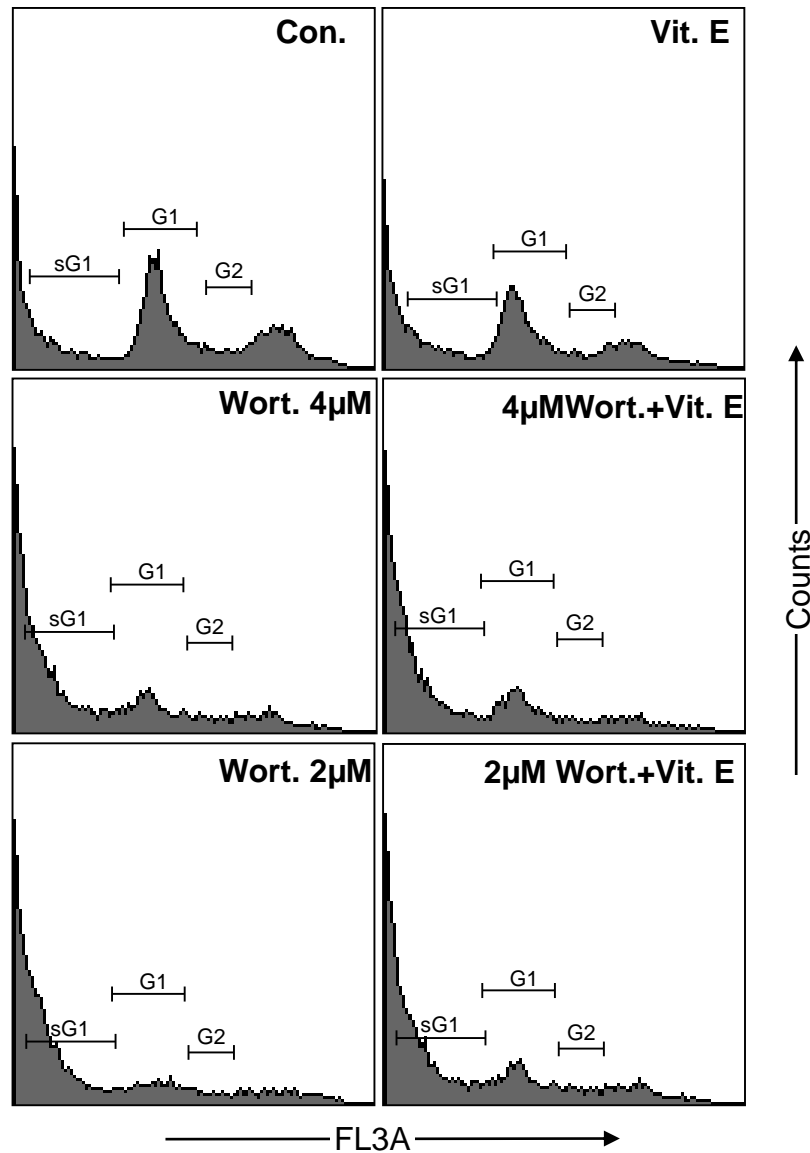

Supplement: S5 Fig — Apoptosis (percentage of sub-G1 cells) was determined by cell cycle analysis in AD-iPSCs pretreated for 2 h with 10 μM alpha-tocopherol (Vit E) and subsequently treated for 24h with 2μM and 4μM wortmannin. Histogram examples of cells treated with wortmannin alone or in combination with Vit E as compared to controls (Con.). Sub-G1 cell populations are indicated (sG1). (PDF) [file pone.0154770.s005.pdf]

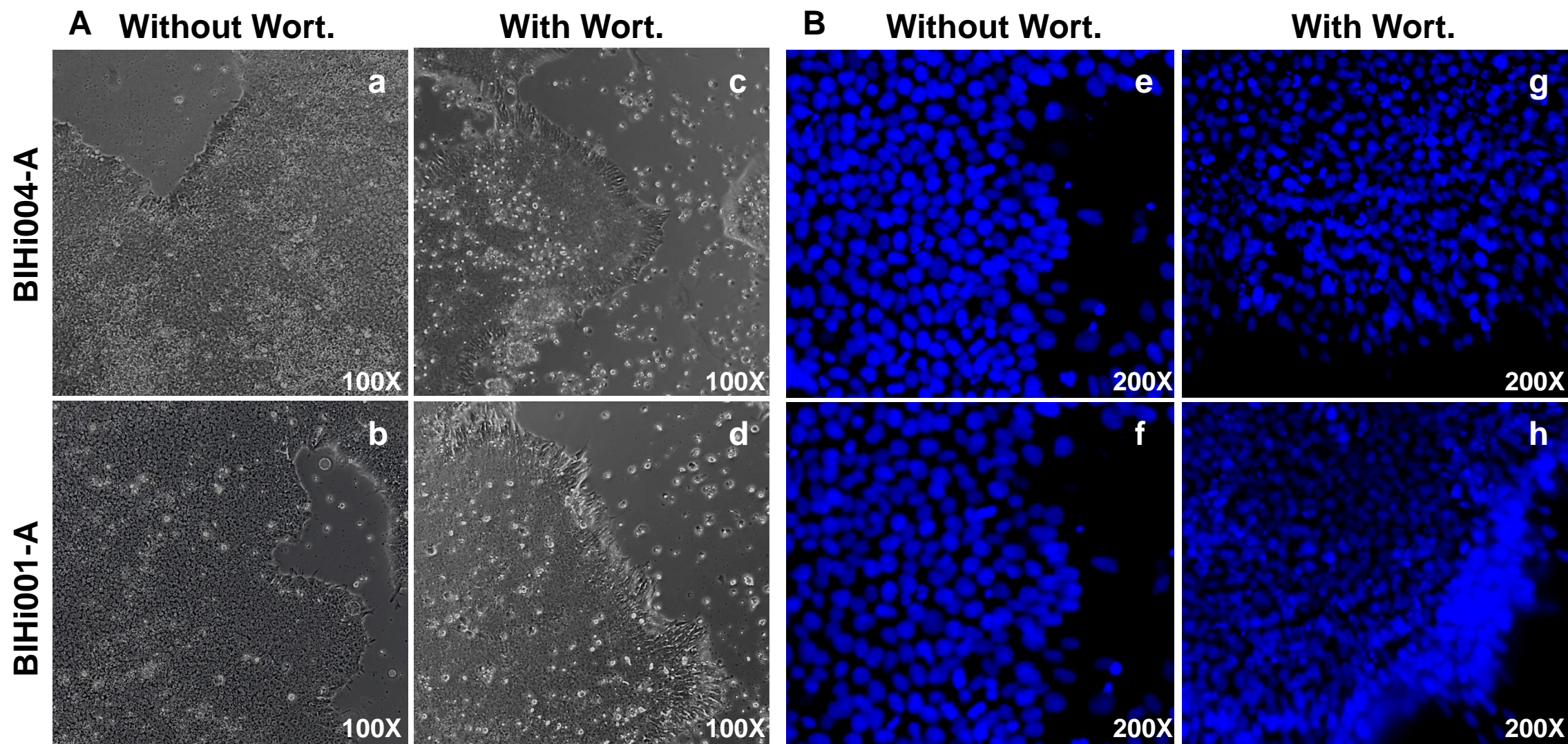

Supplement: S6 Fig — (A) Phase contrast of untreated iPSCs BIHi001-A and BIHi004-A with sharp edges of round colonies (a,b). Untreated iPSC clones show mitotic cells, which can be seen as intense staining, but most of the cells are diffuse blue (e,f). Wortmannin treated iPSCs with frayed edges 2 h after treatment with 4μM wortmannin can be seen in phase contrast (c,d) and as intense blue (g,h). A high proportion of cells showed clear indication of apoptosis by nuclear condensation and fragmentation, particularly pronounced at the edge of the colonies of iPSCs). (PDF) [file pone.0154770.s006.pdf]

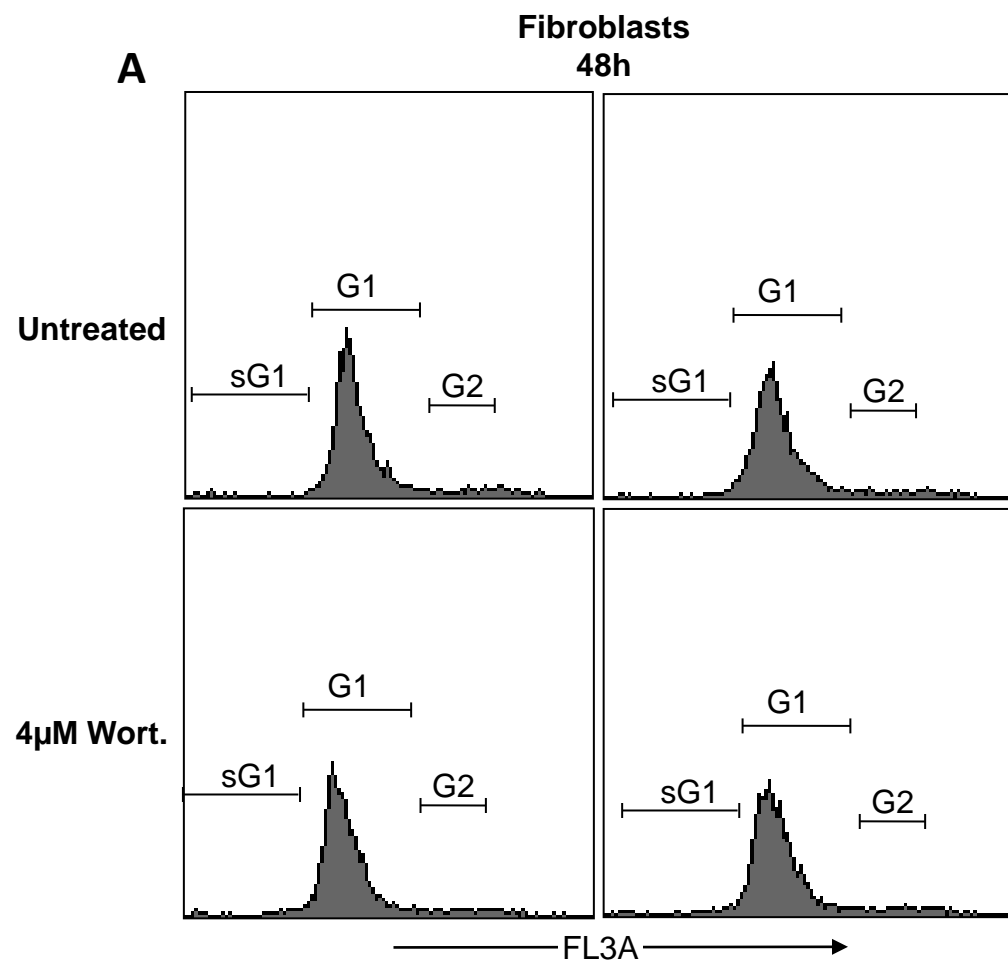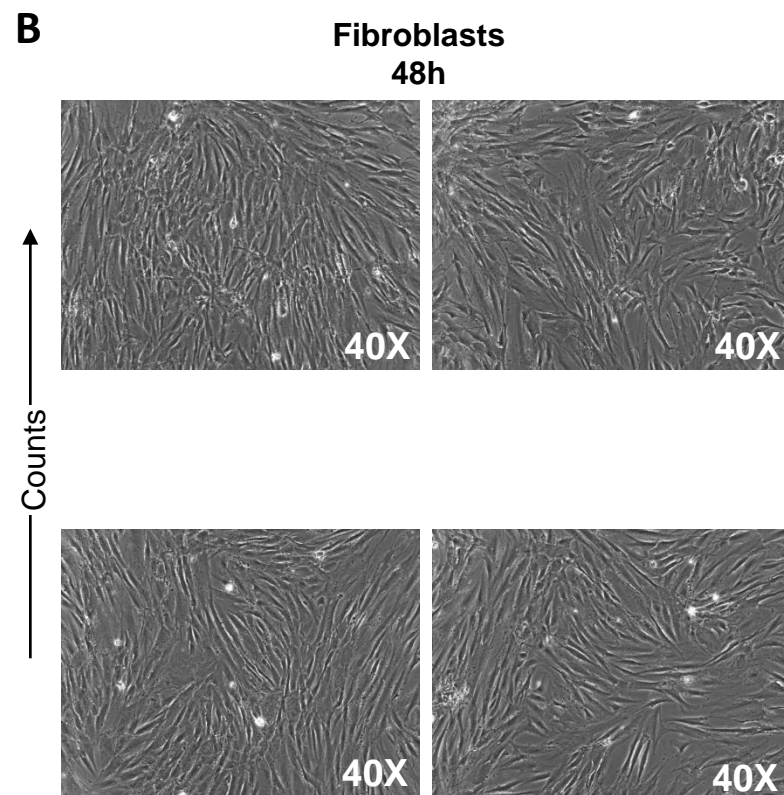

Supplement: S7 Fig — (A) Apoptosis (percentage of sub-G1 cells) was determined by cell cycle analysis in healthy fibroblasts treated for 48h h with 4 μM wortmannin, compared to controls. Sub-G1 cell populations are indicated (sG1). (B) Pictures of fibroblasts were taken after 48 h treatment with 4 μM wortmannin. Magnification 40x. (PDF) [file pone.0154770.s007.pdf]
