# Supplementary material for: PI3K/AKT Signaling Pathway Is Essential for Survival of Induced Pluripotent Stem Cells
Source: PLoS One. 2016 May 3;11(5):e0154770. doi: 10.1371/journal.pone.0154770 (PMC4854383; doi:10.1371/journal.pone.0154770)
Supplement: S2 Fig — Pictures were taken after treatment with 4 μM wortmannin at different times (1 h, 2 h, 6 h, 24 h) of two fibroblast cell lines, corresponding fibroblast derived iPSCs and iPSC-derived neurons. In contrast to both fibroblast cell lines (HFF and NHDF) (A) and iPSC-derived neurons (C), iPSCs BHIi001-A and BHIi004-A (B) showed clear sensitivity to wortmannin induced apoptosis, resulting in a massive cell death with increasing time. The magnifications of all images are 40x. (PDF) [file pone.0154770.s002.pdf]

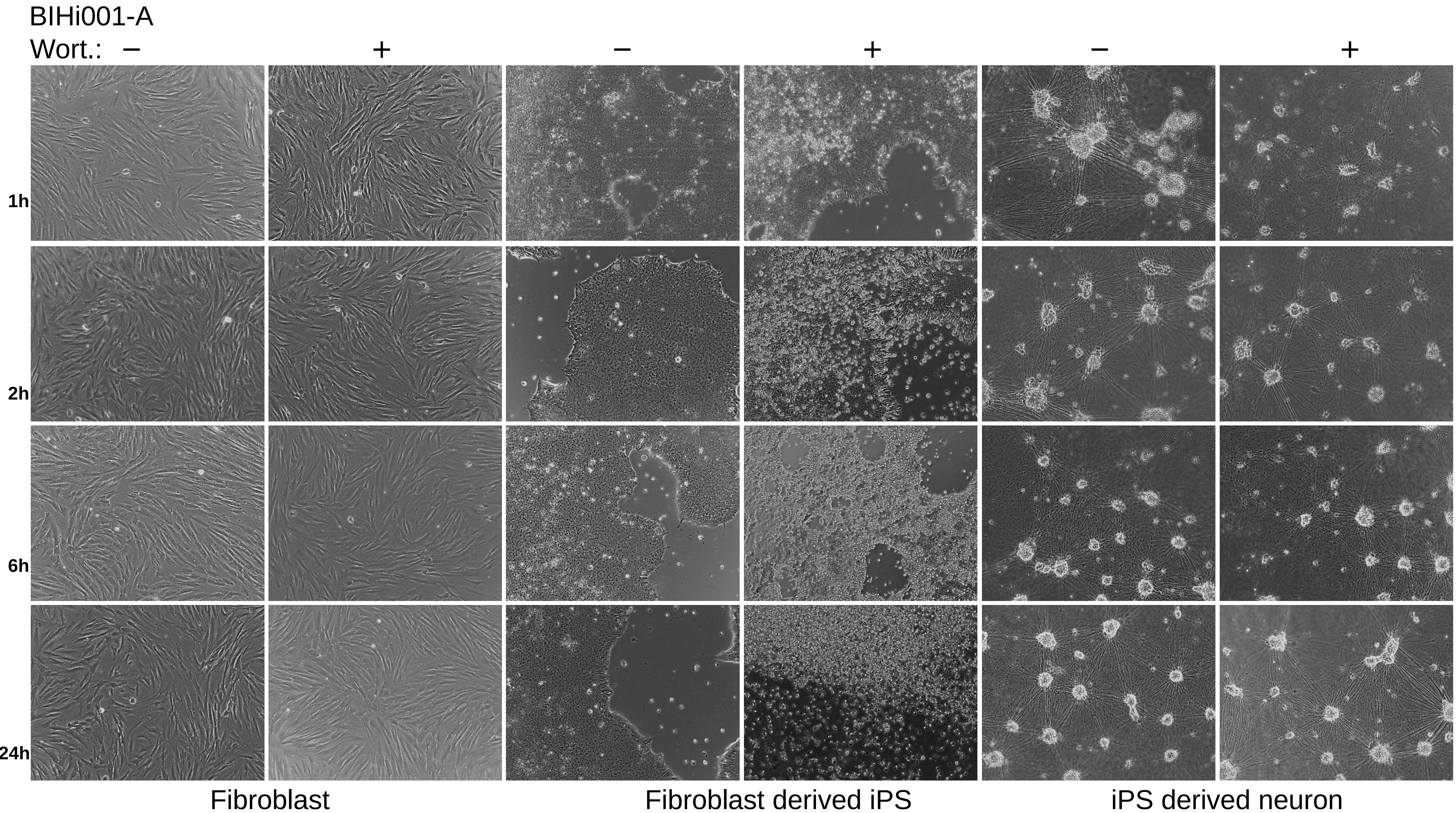
BIHi001-A
Wort.: − + − + − +
1h
2h
6h
24h
Fibroblast
Fibroblast derived iPS
iPS derived neuron

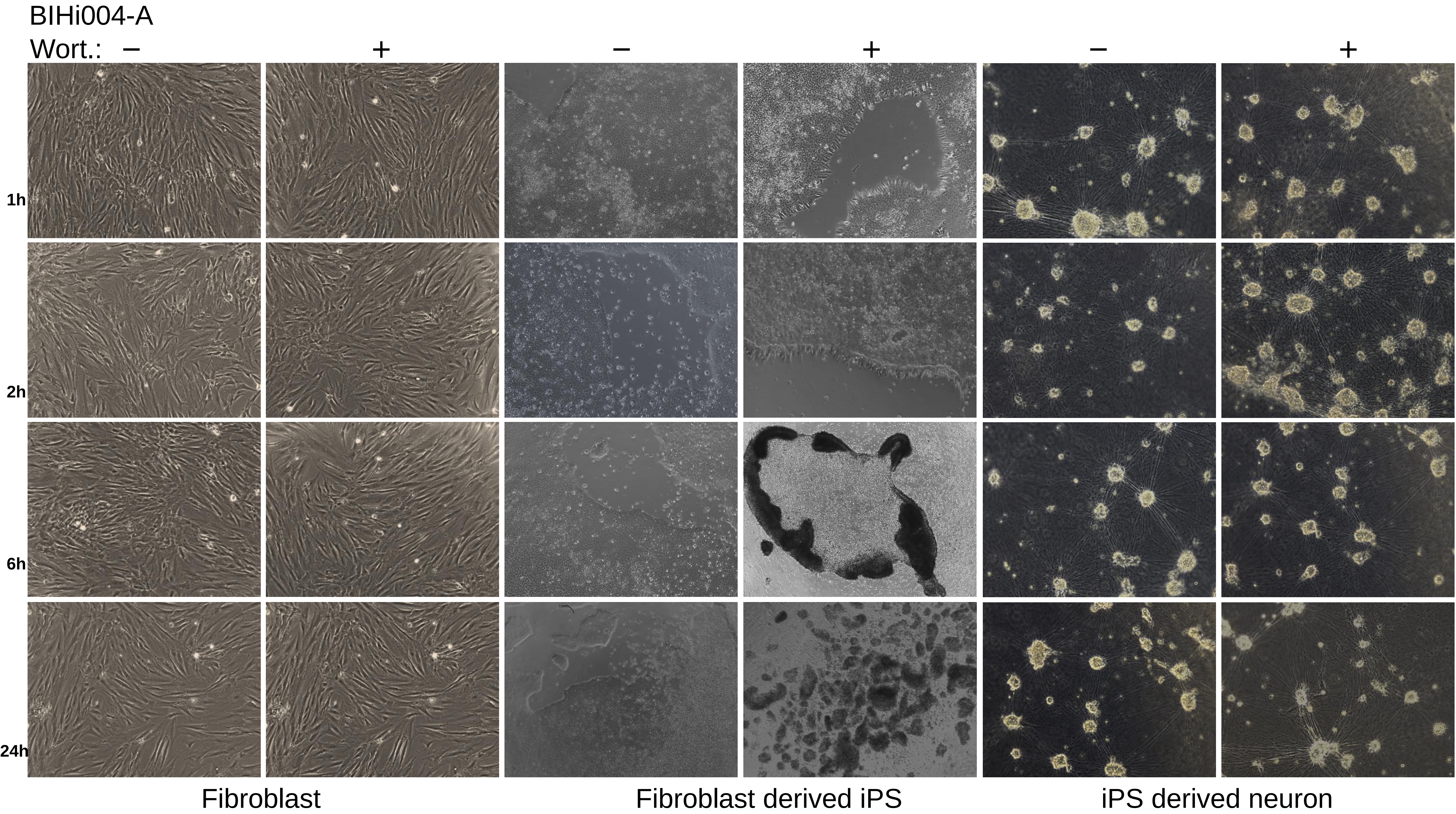
BIHi004-A
Wort.: − + − + − +
1h
2h
6h
24h
Fibroblast
Fibroblast derived iPS
iPS derived neuron
